# The Characteristics of Chemosensory and Opsin Genes in Newly Emerged and Sexually Mature *Agrilus planipennis*, an Important Quarantine Forest Beetle

**DOI:** 10.3389/fgene.2020.604757

**Published:** 2021-01-15

**Authors:** Sifan Shen, Zhizhi Fan, Xun Zhang, Xiangbo Kong, Fu Liu, Zhen Zhang, Xinhua Zhang, Xiumei Hu, Sufang Zhang

**Affiliations:** ^1^Key Laboratory of Forest Protection of National Forestry and Grassland Administration, Research Institute of Forest Ecology, Environment and Protection, Chinese Academy of Forestry, Beijing, China; ^2^College of Grassland Science and Technology, China Agricultural University, Beijing, China; ^3^Forest Control Station of Dawu County, Xiaogan, China

**Keywords:** chemosensory genes, expression levels, opsins, pest management, sexually mature stage

## Abstract

The emerald ash borer (EAB), *Agrilus planipennis*, is a highly destructive quarantine pest. The olfactory and visual systems of *A. planipennis* play different but critical roles at newly emerged and sexually mature stages; however, the molecular basis underlying these differences remain unclear. Consequently, based on deep transcriptome sequencing, we evaluated the expression levels of chemosensory-related proteins and opsins at the two developmental stages of *A. planipennis*. We found 15 new chemosensory-related genes in our transcriptome assembly compared with the previous genome assembly, including 6 that code for odorant-binding proteins (OBPs) and 9 for chemosensory proteins (CSPs). The expression of several chemosensory-related genes (*OBP7, OBP10, CSP1*, and *CSP12*) differed markedly between newly emerged and sexually mature *A. planipennis*. We also found that the expression of *UV opsin 2* and *LW opsin 1* was higher in sexually mature male *A. planipennis*, which may be associated with their strong visual mate detection ability. This study forms the basis for further investigation of the chemosensory and visual system of *A. planipennis*, and these differentially expressed genes between newly emerged and sexually mature stages may serve as targets for the management of this destructive forest pest after sexual maturity.

## Introduction

The emerald ash borer (EAB), *Agrilus planipennis* Fairmaire (Coleoptera: Buprestidae), is native to Asia and eastern Russia, and was not known as a pest before 2004 (Wei et al., 2004). However, since its invasion into the USA and Canada (Cappaert et al., 2005; Poland and McCullough, 2006), this species has caused extensive ash tree (*Fraxinus* spp. L.; Oleaceae) mortality, while its continued spread in North America threatens all native ash species (Wei et al., 2004; Poland and McCullough, 2006), resulting in extensive economic losses. Furthermore, North American ash species planted in Asia were shown to be highly susceptible to *A. planipennis* (Liu et al., 2003). Because larvae feed in phloem and cambial regions of the trees while adults are free-living and feed on the margins of leaves throughout their lifetime, the adult stage is the most conducive for behavioral control of this pest.

Several studies have investigated the chemical ecology of EABs since their potential to cause damage first became apparent (Crook and Mastro, 2010). Emerged *A. planipennis* feed for ~2 weeks before attaining sexual maturity. Host volatiles, such as green leaf volatiles (Poland and McCullough, 2006; Rodriguez-Saona et al., 2006; Groot et al., 2008; Grant et al., 2010) and bark sesquiterpenes (Poland and McCullough, 2006; Crook et al., 2008), can help the freshly emerged EABs locate host plants (Rodriguez-Saona et al., 2006). For instance, (3*Z*)-hexenol, a green leaf volatile, is highly attractive to males (Groot et al., 2008; Grant et al., 2010; Silk et al., 2011). Sexually mature male EABs primarily find females via visual searches (Lelito et al., 2007; Rodriguez-Saona et al., 2007). The males hover 0.3–1.0 m above females, and then rapidly and accurately descend onto them, a behavior known as “paratrooper copulation” (Lelito et al., 2007). However, olfactory cues are more important at short range (≤5 cm) (Pureswaran and Therese, 2009). These observations indicate that both the olfactory and visual systems of adult *A. planipennis* are important for host location and mating activity. Furthermore, host volatiles and trap color were synergistic in attracting EAB to traps suggesting cooperation of the visual and olfactory sensory systems (Crook et al., 2008; Groot et al., 2008). Exploring the mechanisms underlying these two sensory systems is important for controlling adult activities, such as by interfering with their feeding and mating.

Olfaction (Benton et al., 2009; Touhara and Vosshall, 2009; Kaupp, 2010) and vision (Kelber, 1999; Jiggins et al., 2001; Endler and Mappes, 2004) underlie crucial behaviors for insect fitness, such as host and mate location. Semiochemical and visual signal detection is mediated via chemosensory proteins and opsins, respectively. Chemosensory proteins include three types of membrane-bound receptors, two types of binding proteins, and sensory neuron membrane proteins (SNMPs) (Su et al., 2009). Olfactory receptors (ORs) are seven-transmembrane domain proteins with a cytoplasmic N-terminus and extracellular C-terminus, opposite to that seen in vertebrate ORs. These receptors are mainly involved in sensing sex pheromones, host plant volatiles, and other environmental odorants from a distance (Hallem and Carlson, 2006) (Benton et al., 2006; Smart et al., 2008). Each OR forms a heterotetrameric complex with an odorant receptor coreceptor (Orco) (Vosshall and Hansson, 2011). Gustatory receptors (GRs) (Vosshall and Stocker, 2007) are mainly involved in sensing sugar, bitter compounds, and carbon dioxide (Kwon et al., 2007). Antennal ionotropic receptors (IRs) are related to the conserved ionotropic glutamate receptor (iGluR) family, and are expressed in a combinatorial fashion in sensory neurons involved in olfaction and in sensing humidity, salt, and temperature (Croset et al., 2010; Chen et al., 2015; Enjin et al., 2016). The two types of binding proteins comprise small soluble odorant-binding proteins (OBPs) (Vogt, 2003; Sanchez-Gracia et al., 2009) and chemosensory proteins (CSPs) (Pelosi et al., 2006) that primarily bind, solubilize, and transport hydrophobic odor molecules. Some CSP-related genes are also expressed in non-chemosensory tissues and have non-sensory functions (Pelosi et al., 2006). Finally, SNMPs are scavenger proteins of the CD36 family and are associated with pheromone responses (Vogt et al., 2009).

The amino acid sequences of the opsins and chromophores (usually 11-*cis*-retinal) together determine the spectral sensitivity of insect photoreceptors (Gartner and Towner, 1995; Shichida and Imai, 1998; Terakita, 2005). Opsins can be divided into three classes—ultraviolet-sensitive (UV opsins), blue light-sensitive (Blue opsins), and long wavelength-sensitive (LW opsins) — that underpin their sensitivity to ultraviolet (~350 nm), short (~440 nm), and long (~530 nm) wavelength light, respectively (Briscoe, 2001). Insects commonly possess opsins that are sensitive to UV, Blue, and LW spectral peaks (Wakakuwa et al., 2005; Pohl et al., 2009). However, duplications of Blue and LW opsins have been recorded in several insect orders, whereas UV opsin duplications have only been recorded in relatively few species (Lord et al., 2016). In contrast, the loss of opsin genes is mainly found in the Coleoptera (beetles) (Lord et al., 2016).

Mittapalli et al. (2010) was the first to report tissue-specific (midgut and fat body) gene expression in *A. planipennis*, revealing a large number of candidate genes involved in detoxification and providing insights into transcriptionally driven physiological adjustments. Stage-specific unigenes (from larvae, prepupae, pupae, and adults) were also subsequently identified (Duan et al., 2015). Some studies have also focused on the identification of chemosensory genes and opsins in *A. planipennis*. Antennal transcriptome-based identification of odor-processing genes in 2013 yielded 9 OBPs, 2 ORs, and 1 SNMP in *A. planipennis* (Praveen et al., 2013), while a further 2 UV opsins and 3 LW opsins were identified in 2016 (Lord et al., 2016). Genome-wide identification of chemosensory genes led to the annotation of 47 ORs, 30 GRs, 31 IRs, 4 SNMPs, 12 OBPs, and 14 CSPs (Andersson et al., 2019).

After emerging, *A. planipennis* adults usually take 2 weeks to reach sexual maturity, and the sensitivity of the olfactory and visual systems differs between these two stages. By identifying the genes that mediate host plant detection and sex-related activities, more can be learned about the adult stage of this pest, and suitable targets for disrupting feeding and mating can be identified. Here, we compared the head (including antennae) tissue transcriptomes of newly emerged and sexually mature *A. planipennis*. We also increased the depth of sequencing to obtain more information on genes that are expressed at low levels. Our results not only improve the identification of chemosensory genes and opsins in *A. planipennis*, but also provide clues about their function at different adult developmental stages.

## Materials and Methods

### Insects

Sections of ash tree wood containing overwintering *A. planipennis* were collected from the Changping district of Beijing in April 2019. Ash trees infested with *A. planipennis* were identified and the insects were collected from multiple trees. Ash logs with cut ends waxed were placed in cages, maintained in the laboratory at 26 ± 2°C, with 50 ± 10% relative humidity and under a 16:8 h light/dark photoperiod. *Agrilus planipennis* adults emerged ~1 month later, and were collected daily and separated by sex. In order to obtain sexually mature adults, we kept female and male EABs with similar emergence periods in the same glass jar, sealed with gauze, covered with a layer of filter paper, and reared together with clean ash tree leaves collected from the ash tree planted in the Chinese Academy of Forestry. When we found eggs began to appear on the filter paper, we thought adults in this jar have reached sexual maturity. Four groups were established: Eclosion-Female (newly emerged females), Eclosion-Male (newly emerged males), Mating-Females, and Mating-Males. Each live sample contained the heads (including antennae) of 10 insects, and three biological replicates were prepared for each treatment. The samples were immediately frozen in liquid nitrogen and stored at −70°C.

### RNA-Seq Library Preparation and Sequencing

TRIzol reagent (Invitrogen, Carlsbad, CA, USA) was used to extract total RNA as previously described (Zhang et al., 2014, 2017) and the RNA was treated with DNase I to remove genomic DNA (TaKaRa, Dalian, Liaoning, China). The integrity and purity of the total RNA were assessed using a 2100 Bioanalyzer (Agilent Technologies, Inc., Santa Clara, CA, USA); total RNA was quantified using a NanoDrop ND-2000 (Thermo Scientific, Wilmington, DE, USA). High-quality RNA samples with an RIN ≥8.0 were used for sequencing library construction. Sequencing libraries were prepared with 1 μg of total RNA according to the instructions of the Illumina TruSeq™ RNA Sample Preparation Kit (Illumina, San Diego, CA, USA) (Zhulidov et al., 2004; Bogdanova et al., 2008). After quantification using a TBS380 Mini-Fluorometer, the samples were sequenced on an Illumina Hiseq X-Ten Sequencer (Illumina) with a paired-end read length of 150 bp. The biological replicates were sequenced separately.

### *De novo* Assembly of the Sequences

Considering the integrity of gene annotations and the existence of variable splicing, our data assembly does not refer to known genomic data. Clean data were obtained by filtering out adaptors and low-quality reads from raw sequencing data with SeqPrep (https://github.com/jstjohn/SeqPrep) and Sickle (https://github.com/najoshi/sickle) using default parameters. Trinity r20131110 (http://trinityrnaseq.sourceforge.net/) (Grabherr et al., 2011) was used to perform the *de novo* assembly of the clean data, and redundancies were removed using TGICL software (Pertea et al., 2003).

### Annotation

Annotation was performed by blasting the assembled transcripts against seven databases (NR, Swiss-Prot, eggNOG4.5, COG, KOG, GO, and KEGG) to retrieve unigene function annotations (cut-off: 1e-5). BLAST2GO (http://www.blast2go.com/) (Conesa et al., 2005; Götz et al., 2008) was used to search GO annotations for unique transcripts (Ashburner et al., 2000; Krieger et al., 2004), and Kyoto Encyclopedia of Genes and Genomes (KEGG, http://www.genome.jp/kegg/) was used to analyze metabolic pathways.

The genes of interest were then manually identified. Two methods were used for chemosensory-related genes. First, tBLASTx similarity searches were conducted between *A. planipennis* assembled unigenes and chemosensory-related genes from other insects (including *Drosophila melanogaster, Bombyx mori*, and other long-horned beetles) as query sequences (Supplementary Table 1). Secondly, annotated information of the above unigenes was also used. Open reading frames (ORFs) of the candidate genes identified through both methods were then further verified by BLAST.

For the opsin genes, we first performed tBLASTx similarity searches between *A. planipennis* assembled unigenes and opsins from other insects as query sequences (Supplementary Table 2). We also isolated potential light-interacting genes from the transcriptomes by implementing the Phylogenetically-Informed Annotation (PIA) tool (Speiser et al., 2014) in Galaxy. The contigs isolated by the PIA tool were blasted to identify candidate opsins. The candidate genes identified by the two methods were confirmed by ORF blast.

### Gene Expression Quantification

The fragments per kilobase of exon per million mapped reads (FRKM) method was used to calculate the expression level of each transcript (Trapnell et al., 2010). Differentially expressed genes (DEGs) were identified using the EdgeR package in R (http://www.bioconductor.org/packages/2.12/bioc/html/edgeR.html) (Anders and Huber, 2010), and normalization of unigene expression levels and DEGs was performed using the compatible-hits-norm model (Robinson et al., 2010). Statistical tests followed by ANOVA were performed using GraphPad Prism. For olfactory genes, we use asterisks to indicate significant differences between two sexes or two stages, and for opsins, we ues a, b, c to indicate significant differences. The expression levels and *P*-values of differentially expressed genes are summarized in Supplementary Tables 3, 4.

### Phylogenetic Analysis

Phylogenetic analysis of the opsins was performed by MEGA-X, using the *A. planipennis* predicted protein sequences and orthologous genes from other insects (Kumar et al., 2018). The predicted amino acid sequences were aligned using the online version of MAFFT with default settings (https://www.ebi.ac.uk/Tools/msa/mafft/) (Katoh et al., 2005). A 1,000 bootstrap replicated phylogenetic tree was constructed using the Le and Gascuel model (Le and Gascuel, 2008) with frequencies and gamma-distributed sites (LG+F+G) based on the result of MEGA's model test. Tree annotation was performed in Adobe Illustrator.

### Quantification of Gene Expression Levels by Real-Time Quantitative PCR

To obtain the templates for real-time quantitative PCR (qPCR), the same RNA that was used for transcriptome sequencing was reverse transcribed using M-MLV reverse transcriptase (Promega, USA), according to the manufacturer's instructions. We designed qPCR primers to generate 100–250-bp products from the unigene sequences (Supplementary Table 5). The primers were first verified with normal PCR (TaKaRa); the generated amplicons were sequenced to verify the products and ensure that no primer dimers were present. The 2^−ΔΔ*CT*^ method was used to quantify the relative expression level of each gene. The expression levels of all the genes were normalized to that of translation elongation factor 1 alpha (*EF1A*) as previously reported (Zhao et al., 2015a). qPCR was performed in 20-μL reaction volumes (including 10 μL of SuperReal PreMix, Tiangen, Beijing, China) on an ABI7500 thermal cycler (USA) using the following parameters: 2 min at 95 °C, 40 cycles of 20 s at 95°C, 20 s at 58°C, and 20 s at 72°C, and finally 58 to 95°C for melting curve analysis and evaluation of PCR product specificity. Each sample had three technical replicates and three biological replicates.

## Results

### An Overview of the Transcriptomes of *A. planipennis*

More than 10 Gbp of clean data were obtained for each sample by Illumina sequencing, and the Q30 value was higher than 94%. *De novo* assembly of the clean data using Trinity yielded 39,476 contigs with an N50 of 2,291 bp; the length distributions of the transcriptome assemblies are shown in Supplementary Figure 1. The mapping rates of clean data to each sample was >87.68%. Gene annotation against seven databases yielded annotations for 20,767 unigenes.

### Identification of Chemosensory and Opsin Genes in the *A. planipennis* Transcriptomes

We focused our analysis on chemosensory and opsin genes. Although a considerable number of chemosensory genes have been identified in *A. planipennis* at the genome-wide level (Andersson et al., 2019), we identified an additional 15 novel genes in our transcriptome assembly, including 6 that code for OBPs and 9 for CSPs. The 15 new chemosensory genes were submitted to NCBI with the accession numbers MT136965–MT136970 and MT136972–MT136980. Among these 15 genes, only 1 CSP-encoding gene and 1 OBP-encoding gene are in the genome database. Due to the spatiotemporal specificity of the transcriptomic data, we did not detect all the previously identified chemosensory-related genes. For opsin genes, we identified 3 coding for UV opsins, 1 for a UV opsin-like gene, 2 for Green opsins, and 2 for LW opsins. A BLAST comparison indicated that four of the opsin-related genes from our transcriptomic data were new and were not reported by Lord et al. (2016), and these four new *A. planipennis* genes were submitted to NCBI with the accession numbers MT136959–MT136962.

### Characteristics of the Chemosensory-Related Genes From Newly Emerged and Sexually Mature *A. planipennis*

As the olfactory activities of newly emerged (have feeding as the primary behavior) and sexually mature (have mating as the primary behavior) *A. planipennis* are different, it is essential to determine the differences in olfactory responses between these two stages at the molecular level. We compared the expression levels of all the identified chemosensory genes (including previously identified genes and those newly identified in our study) between two developmental stages (newly emerged stage and sexually mature stage) and between the two sexes; several of these genes were verified by qPCR.

**Figure 1:**
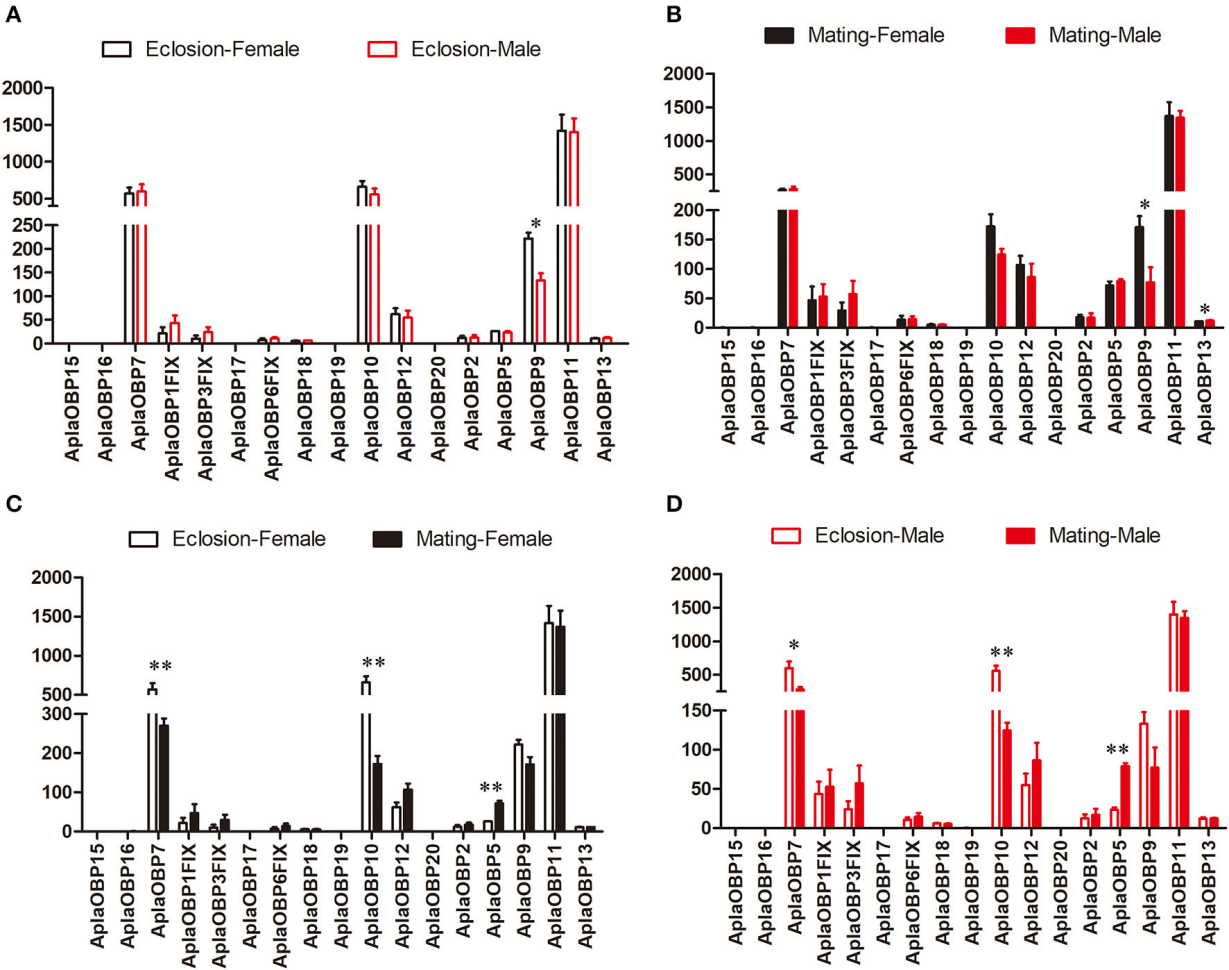
Comparison of the expression levels of OBP genes between the sexes and between two stages. The expression level was determined based on fragments per kb per million reads (FPKM). The standard errors are represented by error bars; different number of asterisks above the bars denote significant differences (*N* = 3, *indicates statistical significance at the 0.05 level, **indicates statistical significance at the 0.01 level). **(A)** Comparison of the expression levels of OBP genes between newly emerged male and female *A. planipennis*. **(B)** Comparison of the expression levels of OBP genes between sexually mature male and female *A. planipennis*. **(C)** Comparison of the expression levels of OBP genes between newly emerged and sexually mature *A. planipennis* females. **(D)** Comparison of the expression levels of OBP genes between newly emerged and sexually mature *A. planipennis* males.

Only *OBP9* and *OBP13* were expressed significantly different between the sexes (Figures 1A,B). The expression of *OBP9* was higher in females than males at both the newly emerged and the sexually mature stages (Eclosion: df = 4, *F* = 19.898, *P* = 0.011; Mating: df = 4, *F* = 9.271, *P* = 0.029), and in males the expression of *OBP13* was higher at sexual maturity than females(df = 4, *F* = 7.177, *P* = 0.044). The expression differences of chemosensory genes between two stages (newly emerged stage and sexually mature stage) were also tested (Figures 1C,D). The expression levels of *OBP7* and *OB10* were significantly higher at the newly emerged stage than at the sexually mature stage in both female (*OBP7*: df = 4, *F* = 19.187, *P* = 0.007; *OBP10*: df = 4, *F* = 47.786, *P* = 0.001; Figure 1C) and male (*OBP7*: df = 4, *F* = 9.267, *P* = 0.038; *OBP10*: df = 4, *F* = 29.516, *P* = 0.006; Figure 1D) *A. planipennis*, while that of *OBP5* was significantly higher at the sexually mature stage in both sexes than at eclosion (Female: df = 4, *F* = 30.407, *P* = 0.003; Male: df = 4, *F* = 137.809, *P* = 0.0003; Figures 1C,D). The expression levels of *OBP7* and *OBP10* were considerably higher than that of *OBP5* in both sexes and stages.

**Figure 2:**
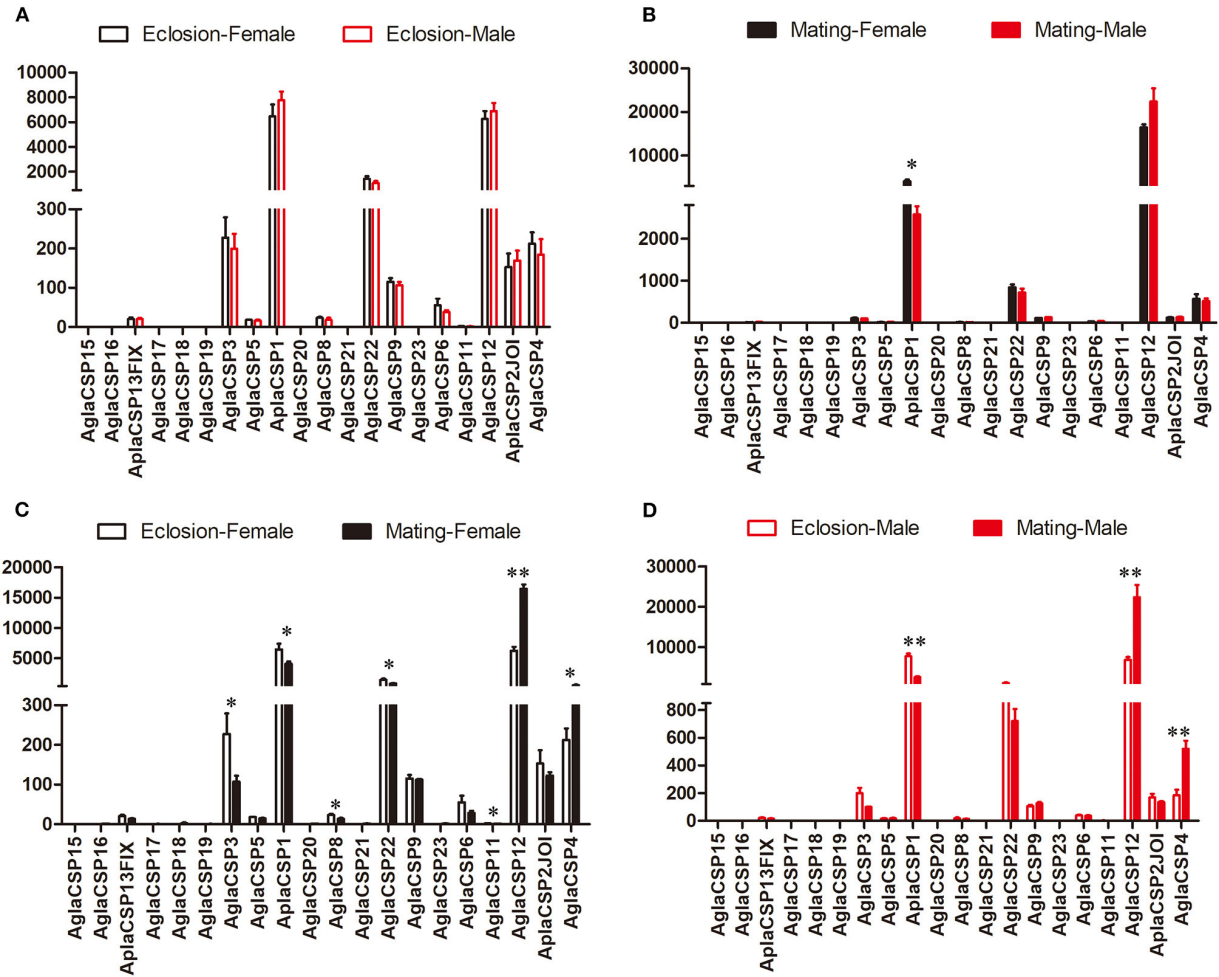
Comparison of the expression levels of CSP genes between the sexes and between two stages. The expression level was determined based on fragments per kb per million reads (FPKM). The standard errors are represented by error bars, different number of asterisks above the bars denote significant differences (*N* = 3, * indicates statistical significance at the 0.05 level, ** indicates statistical significance at the 0.01 level). **(A)** Comparison of the expression levels of CSP genes between newly emerged male and female *A. planipennis*. **(B)** Comparison of the expression levels of CSP genes between male and female *A. planipennis* at sexual maturity. **(C)** Comparison of the expression levels of CSP genes between newly emerged and sexually mature *A. planipennis* females. **(D)** Comparison of the expression levels of CSP genes between newly emerged and sexually mature *A. planipennis* males.

The expression patterns of the CSPs, which are also binding protein, also showed few differences between sexes, in contrast to that observed between the two stages (Figure 2). Only *CSP1* showed higher expression in female *A. planipennis* at sexual maturity when compared with that of sexually mature males (df = 4, *F* = 9.083, *P* = 0.030; Figure 2B) (no differences in CSP expression were found at the newly emerged stage; Figure 2A). However, in female *A. planipennis*, seven CSPs were differentially expressed between the two stages (Figure 2C). Of these, *CSP3, CSP1, CSP8, CSP22*, and *CSP11* exhibited higher expression at the newly emerged stage(*CSP3*: df = 4, *F* = 6.593, *P* = 0.05; *CSP1*: df = 4, *F* = 6.800, *P* = 0.048; *CSP8*: df = 4, *F* = 9.819, *P* = 0.026; *CSP22*: df = 4, *F* = 8.288, *P* = 0.035; *CSP11*: df = 4, *F* = 8.761, *P* = 0.032), while the expression levels of *CSP12* and *CSP4* were higher at the sexually mature stage(*CSP12*: df = 4, *F* = 115.158, *P* = 0.0001; *CSP4*: df = 4, *F* = 7.329, *P* = 0.042). In male *A. planipennis*, three CSPs were differentially expressed between the two stages, and showed a similar pattern to that of females (Figure 2D). Among the DEGs, *CSP1* was highly expressed at eclosion (df = 4, *F* = 52.156, *P* = 0.002)and *CSP12* was highly expressed at mating(df = 4, *F* = 24.836, *P* = 0.008), whereas the other CSPs (*CSP3, CSP8, CSP22*, and *CSP4*) showed low abundance.

**Figure 3:**
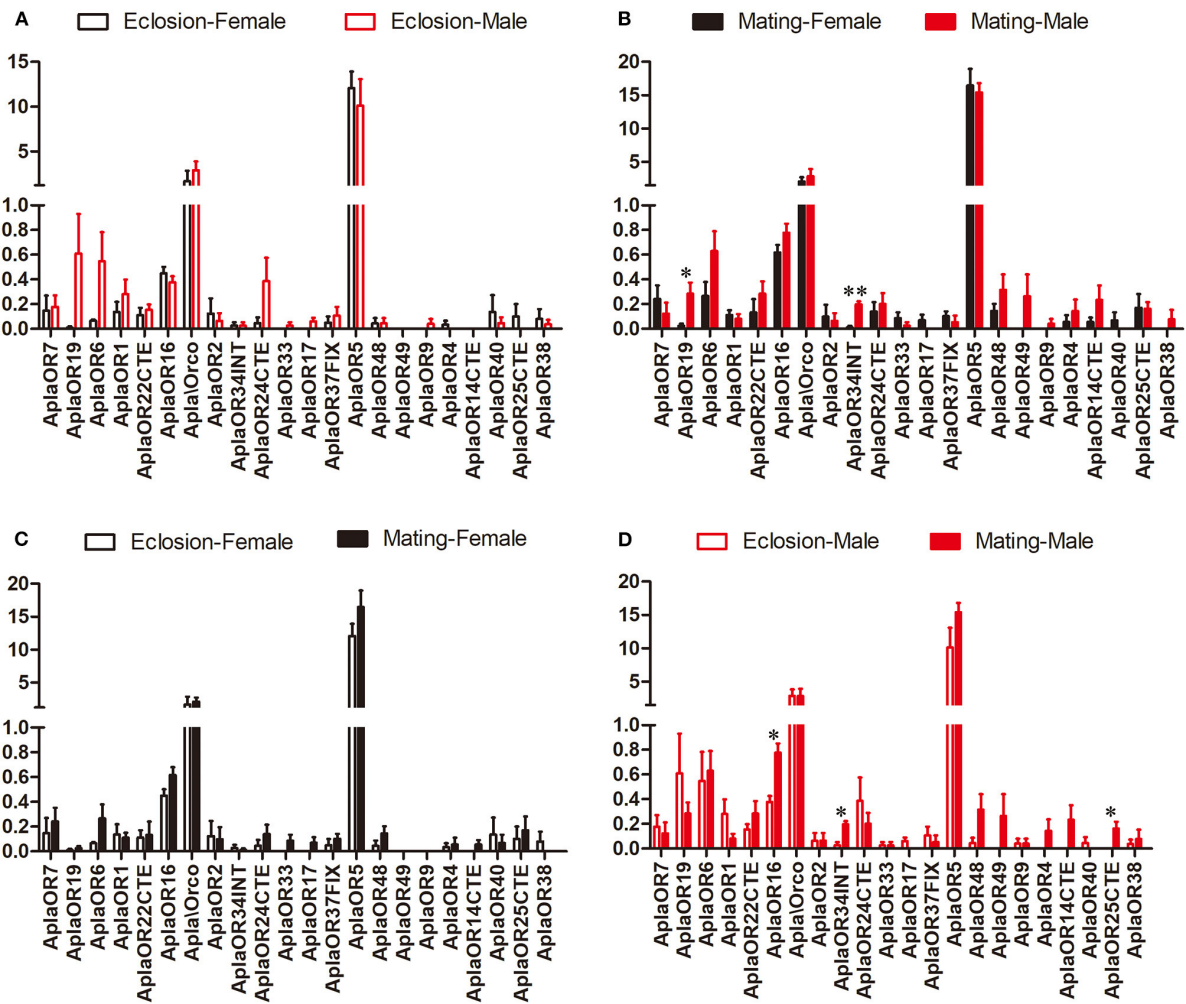
Comparison of the expression levels of OR genes between the sexes and between two stages. The expression level was determined based on fragments per kb per million reads (FPKM). The standard errors are represented by error bars, different number of asterisks above the bars denote significant differences (*N* = 3, * indicates statistical significance at the 0.05 level, ** indicates statistical significance at the 0.01 level). **(A)** Comparison of the expression levels of OR genes between newly emerged male and female *A. planipennis*. **(B)** Comparison of the expression levels of OR genes between male and female *A. planipennis* at sexual maturity. **(C)** Comparison of the expression levels of OR genes between newly emerged and sexually mature *A. planipennis* females. **(D)** Comparison of the expression levels of OR genes between newly emerged and sexually mature *A. planipennis* males.

The expression of the ORs, important *A. planipennis* receptor proteins, was compared between the two stages and between the sexes. No DEGs between the sexes were found in newly emerged *A. planipennis* (Figure 3A). *OR19* and *OR34INT* was differentially expressed between the sexes at sexual maturity (*OR19*: df = 4, *F* = 10.882, *P* = 0.022; *OR34INT*: df = 4, *F* = 47.336, *P* = 0.001); however, their expression levels were low (Figure 3B). *OR5* showed the highest expression level among the ORs without obvious difference both between the sexes and between the two stages. No DEGs between two stages were found in females (Figure 3C). *OR16* and *OR34INT* was differentially expressed between the two stages in males (*OR16*: df = 4, *F* = 19.862, *P* = 0.011; *OR34INT*: df = 4, *F* = 20.320, *P* = 0.011); however, their expression levels were very low (Figure 3D).

The expression patterns of other chemosensory genes (GRs, IRs, and SNMPs) were also analyzed, and, overall, differed little between the two stages and between the sexes (Supplementary Figures 2–4). Among the GRs, only *GR8NTE*, the expression level of which was very low, was differentially expressed between the sexes at sexual maturity (df = 4, *F* = 8.344, *P* = 0.034, Supplementary Figure 2B), and also between the two stages in females (df = 4, *F* = 49.799, *P* = 0.001; Supplementary Figure 2C). Among the IRs, the expression of *IR76b* was higher in newly emerged males (Supplementary Figure 3A) and that of *IR93a* and *IR41aINT* differed between the sexes at sexual maturity (*IR93a*: df = 4, *F* = 7.469, *P* = 0.041; *IR41aINT*: df = 4, *F* = 7.366, *P* = 0.042; Supplementary Figure 3B). Notably, IRs were the only class of chemosensory genes that differed between the sexes but not between the two stages. Finally, no significant differences in SNMP expression were detected either between the two stages or between the sexes (Supplementary Figure 4). We selected three DEGs related to olfaction and vision in *A. planipennis* (*OBP7, UV opsin 2*, and *LW opsin 1*) and some non-differentially expressed genes for verification by qPCR, with the results indicating that the transcriptome data were reliable (Supplementary Figure 5).

### Phylogenetic Analysis of the Opsins From *A. planipennis*

**Figure 4:**
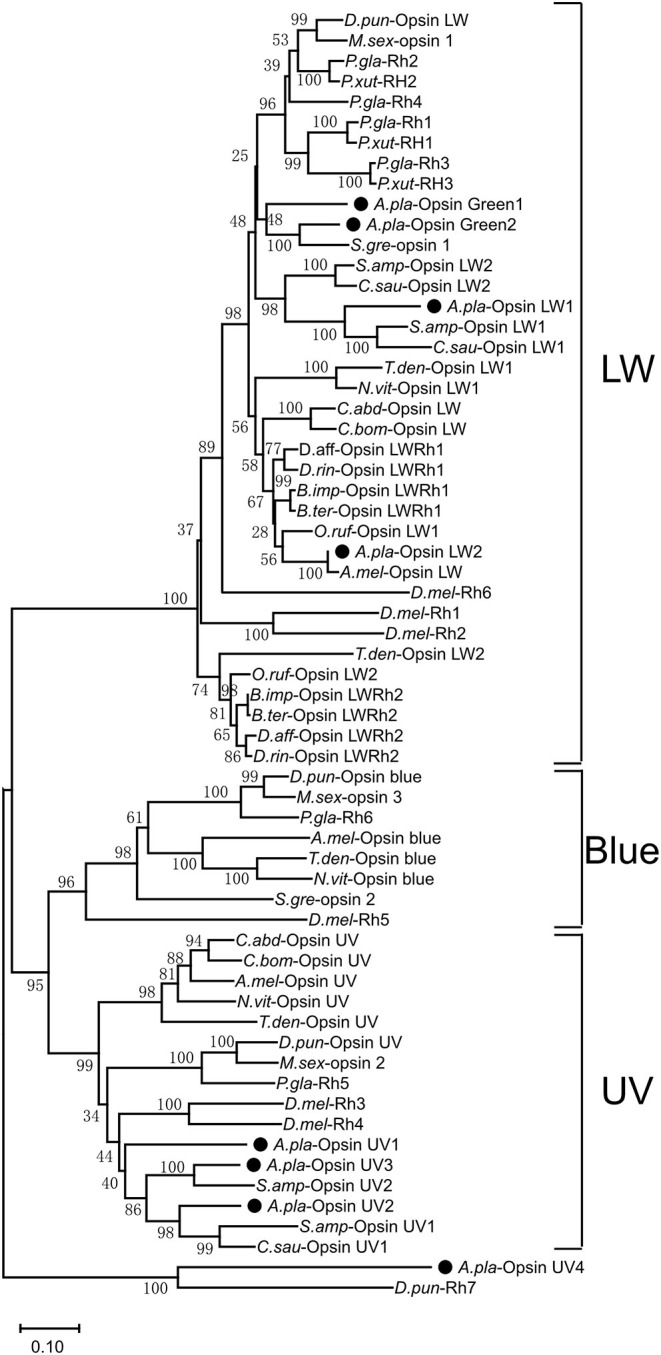
Phylogenetic analysis of *A. planipennis* opsins and those of other insects. The phylogenetic tree was constructed with the sequences of opsin proteins from *A. planipennis* and other species, including the model insects *D. melanogaster* and *Apis mellifera*, as well as other insects from Lepidoptera, Hymenoptera, and Coleoptera.

A phylogenetic tree was constructed with opsin protein sequences from *A. planipennis* and other species, including the model insects *D. melanogaster* and *A. mellifera*, as well as other insects from the orders Lepidoptera, Hymenoptera, and Coleoptera (Figure 4). The phylogenetic analysis showed that UV and LW opsins from *A. planipennis* were clustered into the corresponding branches of the other insects, and no Blue opsins were found in *A. planipennis*. The UV-like opsin gene of *A. planipennis* was clustered with a circadian photoreceptor (Rh7) from *D. melanogaster* and other insects in a separate branch.

### The Expression Levels of the Opsins in Newly Emerged and Sexually Mature *A. planipennis*

**Figure 5:**
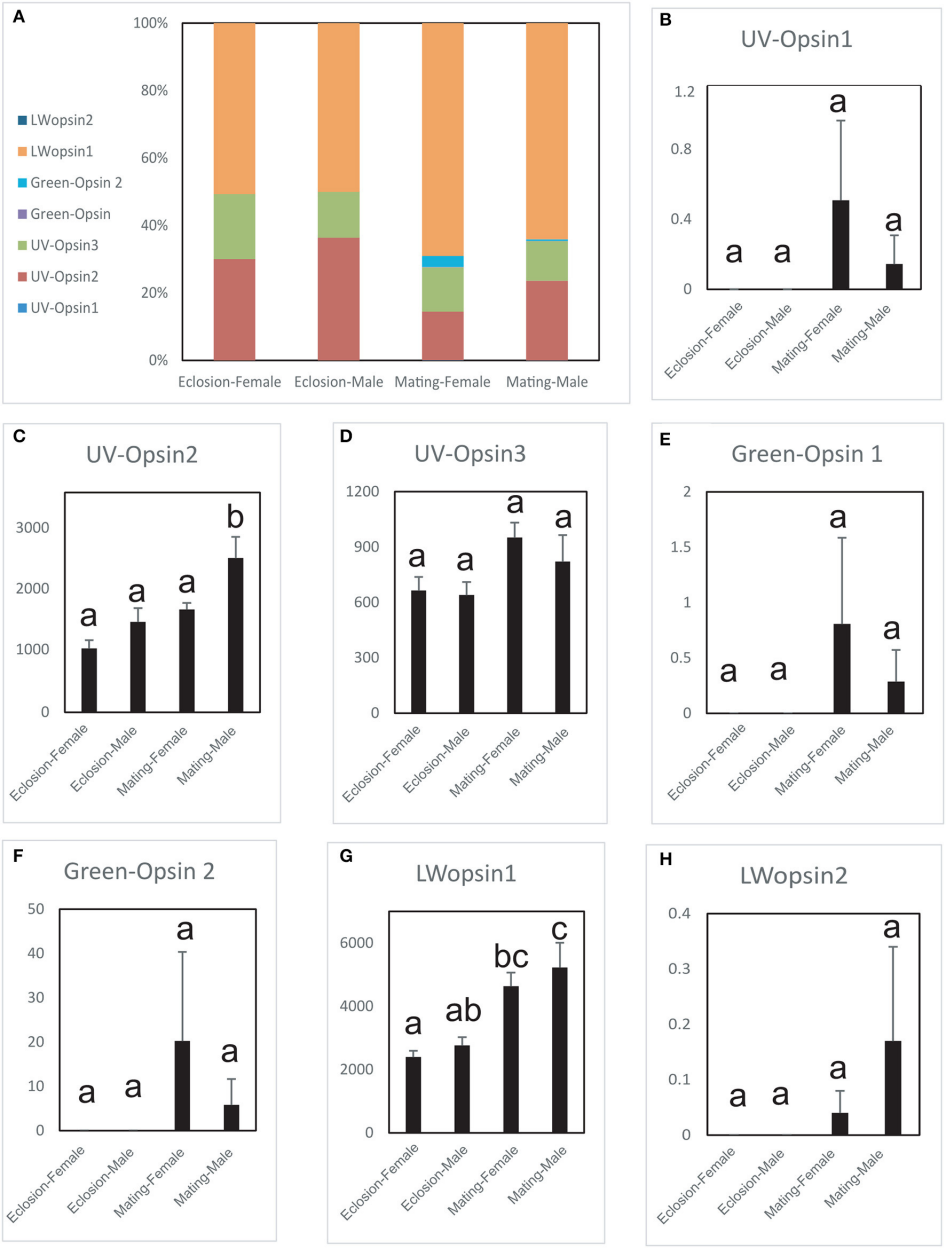
Comparison of opsin expression levels between the sexes and between two stages. The expression levels were determined based on fragments per kb per million reads (FPKM). The standard errors are represented by error bars, different lowercase letters (a, b, c) above the bars denote significant differences (*N* = 3). **(A)** The proportions of the expression levels of different genes in the different sexes and stages. Comparison of the expression levels of *UV opsin 1* **(B)**, *UV opsin 2* **(C)**, *UV opsin 3* **(D)**, *Green opsin 1* **(E)**, *Green opsin 2* **(F)**, *LW opsin 1* **(G)**, and *LW opsin 2* **(H)** between the sexes and two stages (newly emergent and sexually mature).

The expression levels of the seven opsin genes differed between newly emerged and sexually mature *A. planipennis* (Figure 5). Among the seven opsins, *UV opsin 1* (Figure 5B), *UV opsin 3* (Figure 5D), *Green opsin 1* (Figure 5E), *Green opsin 2* (Figure 5F), and *LW opsin 2* (Figure 5H) showed no differences in expression either between the sexes or between the two developmental stages. The expression of *UV opsin 2* (df = 4, *F* = 12.164, *P* = 0.002; Figure 5C) and *LW opsin 1* (df = 4, *F* = 8.338, *P* = 0.006; Figure 5G) was significantly higher in sexually mature males, comparing with the females. Additionally, three of the seven opsins were highly expressed (*UV opsin 2, UV opsin 3*, and *LW opsin 1*). The proportions of gene expression levels in newly emerged and sexually mature *A. planipennis* of both sexes are illustrated in Figure 5A. The ratio of LW opsins was higher at the sexually mature stage than in the newly emerged stage; the opposite result was observed for the ratio of UV opsins.

## Discussion

Although Coleoptera is the largest order of insects, relatively few studies have investigated their sensory systems when compared with Diptera, Lepidoptera, and Hymenoptera (Engsontia et al., 2014; Zhao et al., 2015b). Chemosensory genes have been identified in several Coleoptera insects (Mitchell et al., 2020). In this study, we selected two different *A. planipennis* developmental stages—the newly emerged stage and the sexual maturity—to identify and compare the key proteins involved in the chemosensory and vision systems of this species.

Because OBPs are involved in the first step of odor detection; therefore, they are the focus of olfactory research (Pelosi et al., 2006). Based on previous results, we sought to identify additional OBPs in *A. planipennis*, and found six new OBP-coding genes. We found that their expression was largely similar between sexes, both in newly emerged and sexually mature *A. planipennis*. However, two OBPs (*OBP7* and *OBP10*) were highly expressed, and both showed markedly higher expression at the newly emerged stage. Studies have indicated that host volatiles, including green leaf volatiles, and especially (3Z)-hexenol (Rodriguez-Saona et al., 2007; Groot et al., 2008; Grant et al., 2010; Ryall et al., 2012), and bark sesquiterpenes (Crook et al., 2008), can help newly emerged *A. planipennis* locate host trees. *OBP7* and *OBP10* of *A. planipennis* may be associated with the sensing of these host volatiles; however, further functional studies are needed to determine this.

The CSPs, comprising another class of small binding proteins, also showed few between-sex differences in adult *A. planipennis*. However, two CSPs were found to be significantly highly expressed: the expression of *CSP1* was higher at the newly emerged stage, while that of *CSP12* was higher at sexual maturity for both sexes. The function of these highly expressed genes is still unclear, and in the future, we will perform functional analysis to determine their functions.

OR proteins are key receptors in the olfaction system, translating chemical signals (such as those from host volatiles and pheromones) into electrical nerve impulses (Clyne et al., 1999; Vosshall et al., 1999). Our results showed that only two low expression ORs (*OR16* and *OR34INT*) expressed significantly higher in the mature males, while no difference were found for *OR5*, which displayed the strongest expression among the ORs. The function of these gene need further studies.

We did not find Blue opsins in *A. planipennis*, as also previously reported (Lord et al., 2016). However, three UV opsins and four LW opsins (including Green opsins and LW opsins) were found to be duplicated, more than that previously reported (Lord et al., 2016). Interestingly, two opsins (*UV opsin 2* and *LW opsin 1*) showed markedly higher expression in sexually mature males, and the proportion of expressed LW opsins was higher at the sexually mature stage than at the newly emerged stage. Sexually mature male *A. planipennis* are known to have strong visual mate searching ability (Lelito et al., 2007; Crook et al., 2009), and higher expression levels of these opsin genes may be related to this characteristic.

Overall, the expression levels of chemosensory genes in *A. planipennis* were largely similar between the sexes at both adult stages. However, the expression levels of several chemosensory genes (*OBP7, OBP10, CSP1*, and *CSP12*) were significantly different between newly emerged and sexually mature adult *A. planipennis*, and *OBP7, OBP10, CSP1* exhibited high levels of expression at eclosion, *CSP12* exhibited high levels of expression at mating stage. For the vision genes, the expression of *UV opsin 2* and *LW opsin 1* was higher in sexually mature *A. planipennis* males. Studies on the functions of these chemosensory-related genes and opsins are urgently needed. In the future, we will use RNAi and electrophysiological technology, combined with behavioral tests to perform functional verification, and discuss the physiological processes that these genes may participate in.

During sampling, the whole head was selected for sequencing due to the small antennae of the adults and the large sample demand. However, this also brought some problems: the head portion majorly represents chemosensory/olfactory and visual related functions, we do understand and acknowledge the fact that this complex portion of the body expresses other genes involved in other vital physiological processes. Our work forms the basis for further investigation of the functional mechanisms underlying the chemosensory and visual systems in *A. planipennis*, while the chemosensory and opsin genes identified as being differentially expressed between newly emerged and sexually mature *A. planipennis* may serve as novel targets for the management of this destructive forest pest.

## Data Availability Statement

Raw reads from sequencing are deposited in the Sequence Read Archive (SRA) database with NCBI accession SRR11309616- SRR11309628.

## Author Contributions

SZ designed the experiments, analyzed the data, and drafted the manuscript. XiZ and XH supplied the insects. ZZ revised the manuscript. XK and FL helped with insect feeding. XuZ helped with the experimental techniques. SS and ZF performed the laboratory experiments and revised the manuscript. All authors contributed to the article and approved the submitted version.

## Conflict of Interest

The authors declare that the research was conducted in the absence of any commercial or financial relationships that could be construed as a potential conflict of interest.

## Supplementary Material

Supplementary Figure 1 Length distribution of unigenes obtained in *A. planipennis* head (including antennae) transcriptome.

Supplementary Figure 2 Expression levels of GR genes between different sexes and stages.

Supplementary Figure 3 Expression levels of IR genes between different sexes and stages.

Supplementary Figure 4 Expression levels of SNMP genes between different sexes and stages.

Supplementary Figure 5 Validation of gene expression levels in *A. planipennis* with qPCR.

Supplementary Table 1 Accession numbers of chemosensory genes from other insects used for chemosensory gene identification in *A. planipennis*.

Supplementary Table 2 Accession numbers of opsins from other insects used for opsin gene identification in *A. planipennis*.

Supplementary Table 3 The expression levels and significance of differentially expressed genes (DEGs) in female *A. planipennis*.

Supplementary Table 4 The expression levels and significance of differentially expressed genes (DEGs) in male *A. planipennis*.

Supplementary Table 5 Primers used for validation of RNA-seq data by qPCR.
