# Supplementary figures and images for: The Characteristics of Chemosensory and Opsin Genes in Newly Emerged and Sexually Mature Agrilus planipennis, an Important Quarantine Forest Beetle
Source: Front Genet. 2021 Jan 15;11:604757. doi: 10.3389/fgene.2020.604757 (PMC7844324; doi:10.3389/fgene.2020.604757)

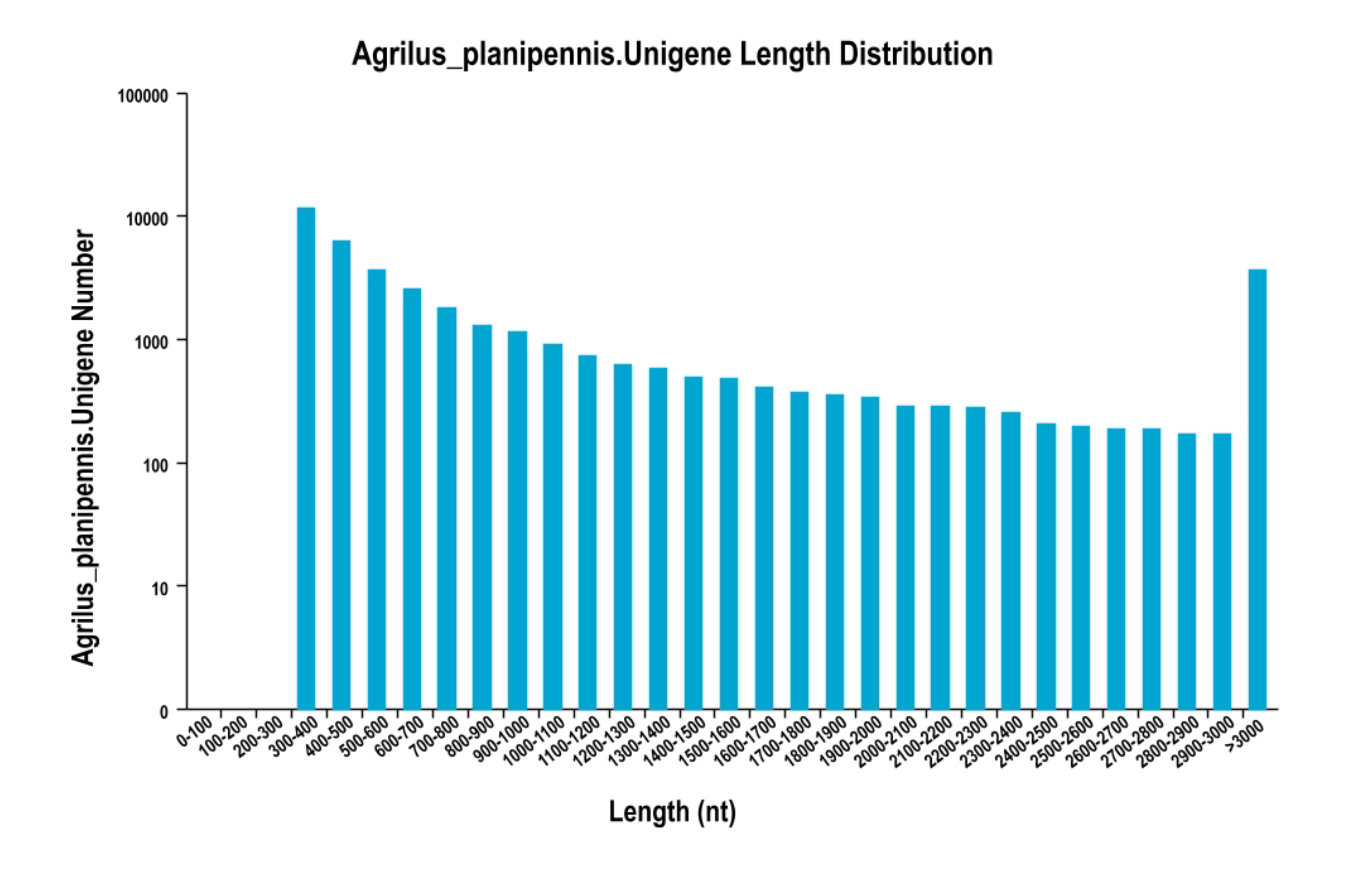

Supplement: Supplementary Figure 1 — Length distribution of unigenes obtained in A. planipennis head (including antennae) transcriptome. [file Image_1.tif]

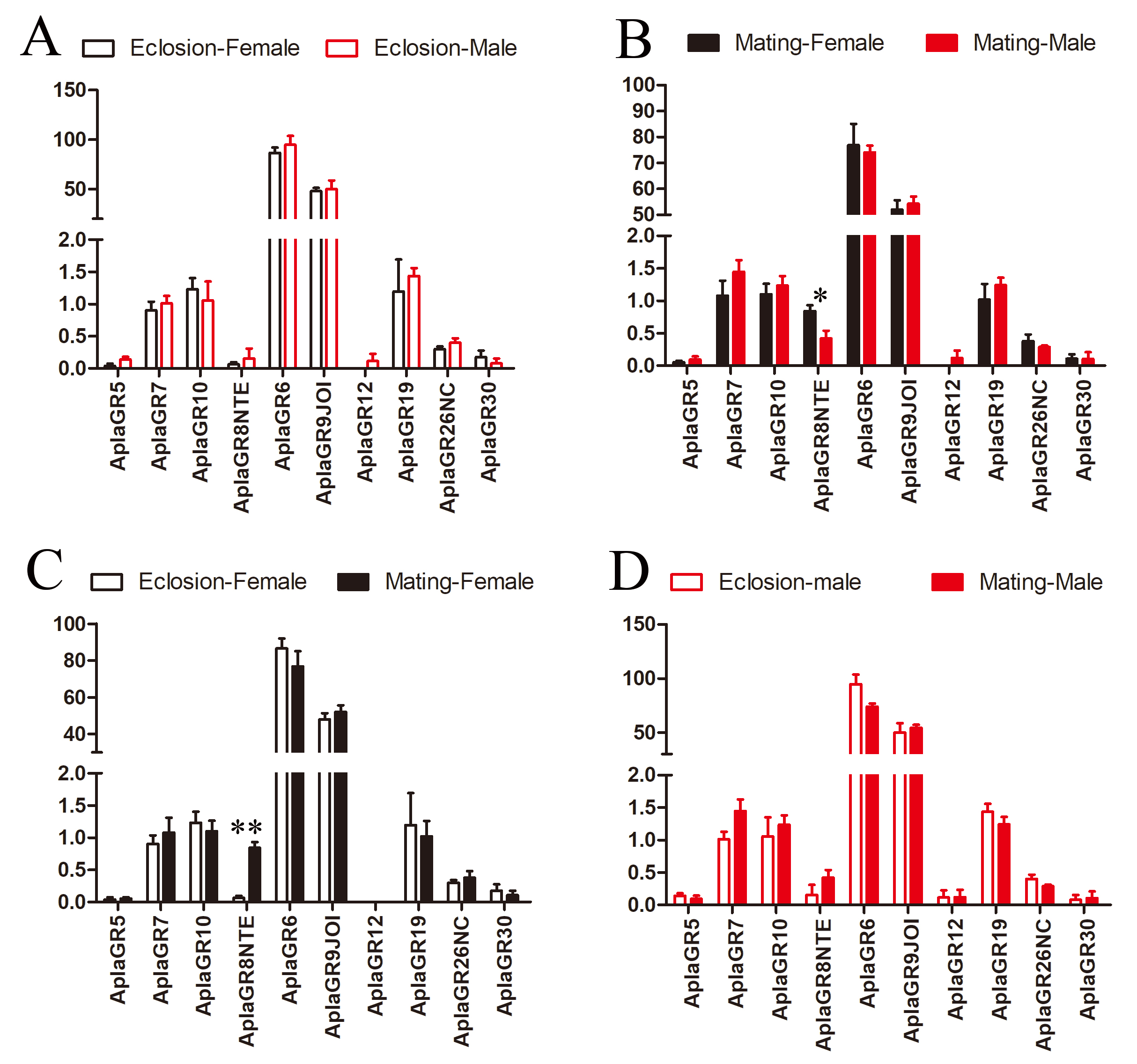

Supplement: Supplementary Figure 2 — Expression levels of GR genes between different sexes and stages. [file Image_2.jpg]

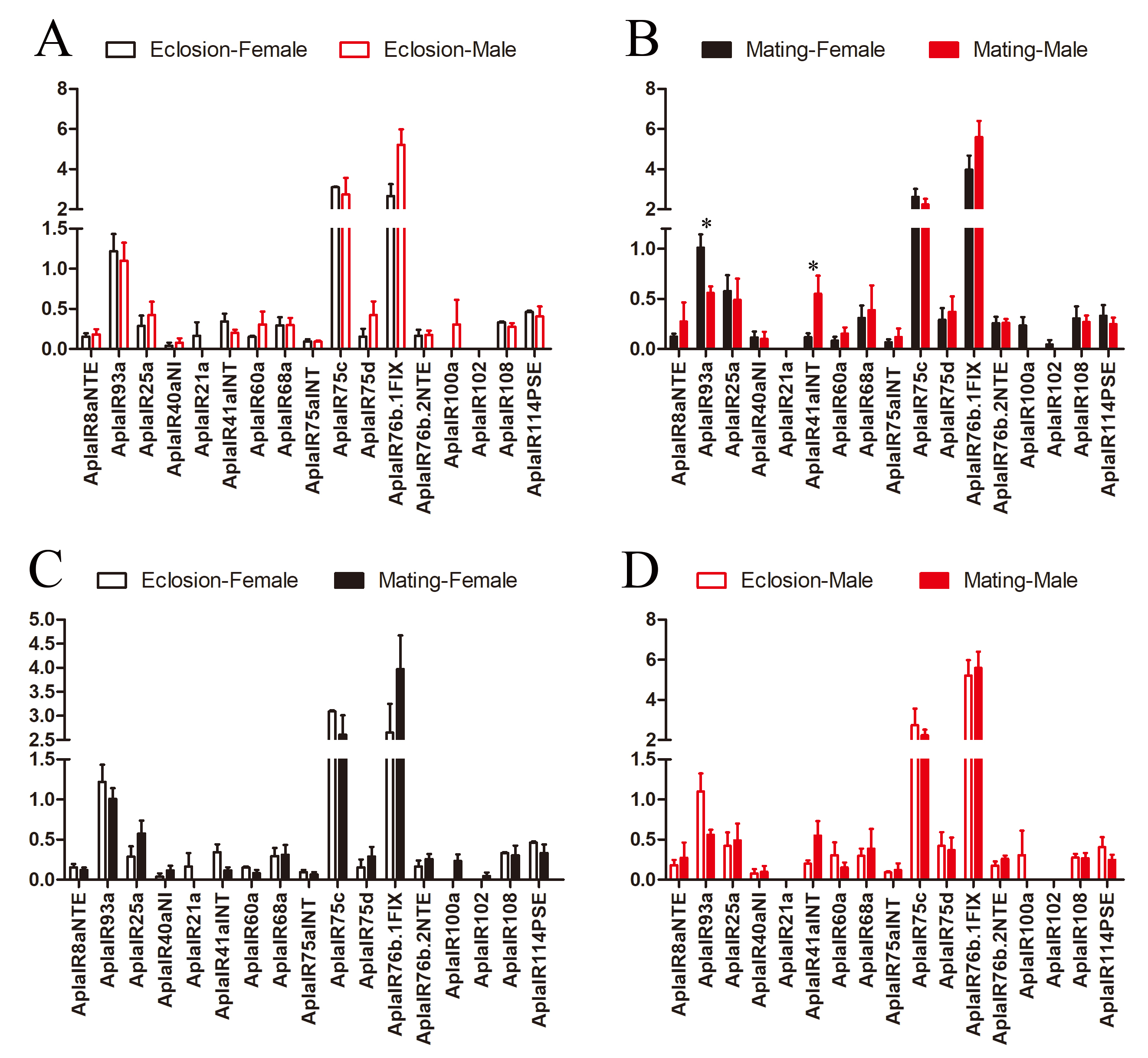

Supplement: Supplementary Figure 3 — Expression levels of IR genes between different sexes and stages. [file Image_3.jpg]

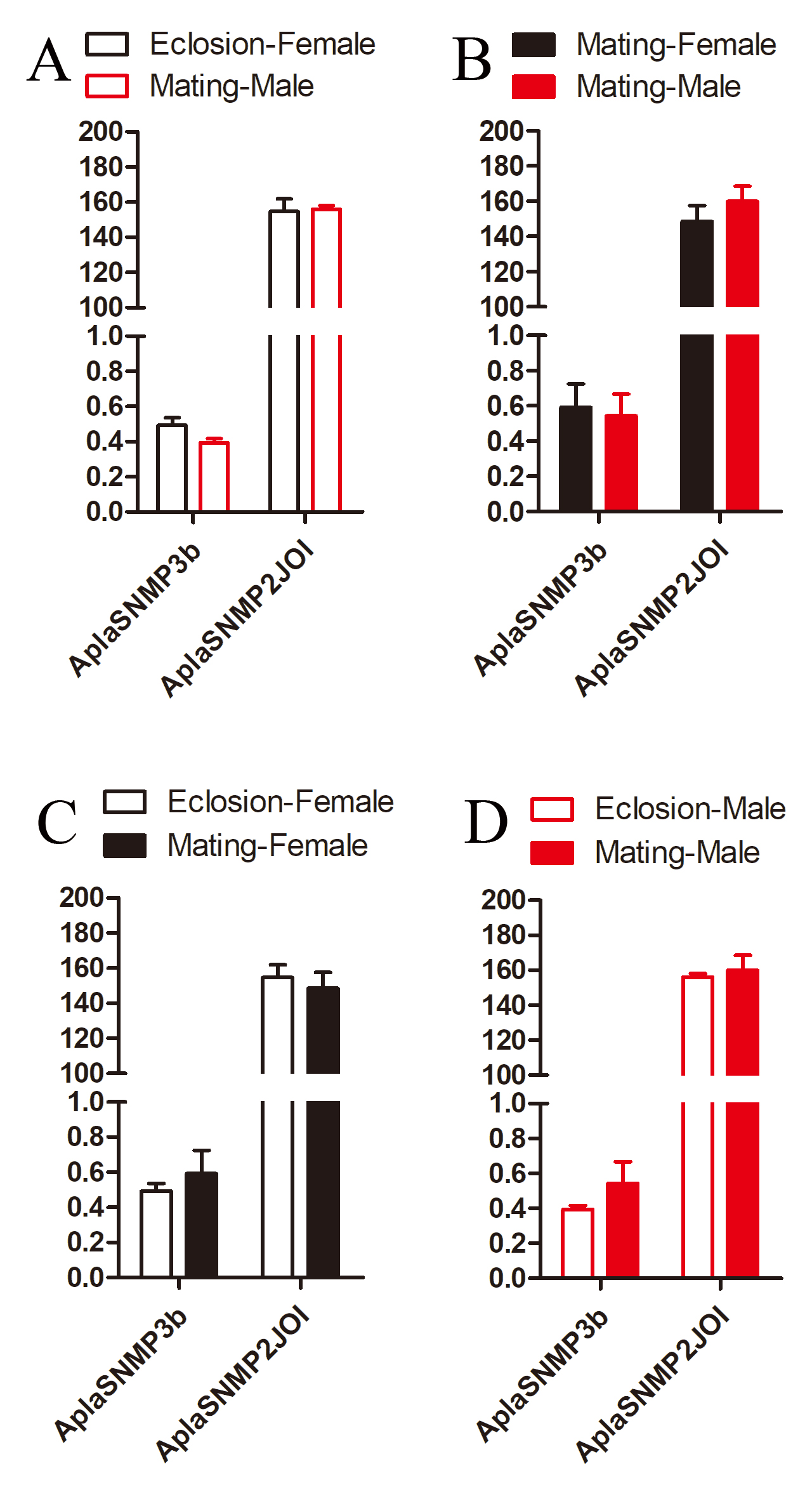

Supplement: Supplementary Figure 4 — Expression levels of SNMP genes between different sexes and stages. [file Image_4.jpg]

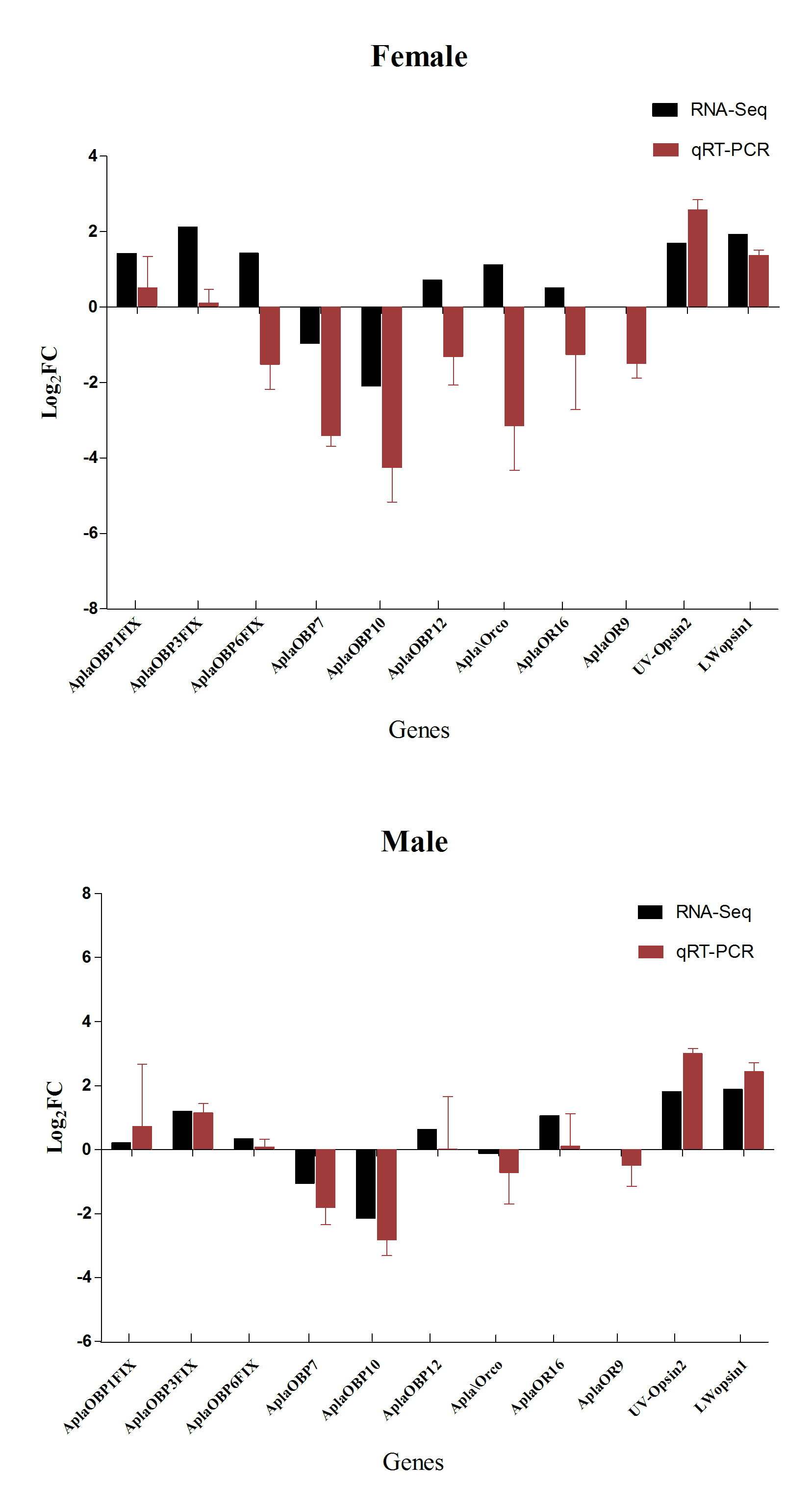

Supplement: Supplementary Figure 5 — Validation of gene expression levels in A. planipennis with qPCR. [file Image_5.tif]
