# Supplementary material for: The Characteristics of Chemosensory and Opsin Genes in Newly Emerged and Sexually Mature Agrilus planipennis, an Important Quarantine Forest Beetle
Source: Front Genet. 2021 Jan 15;11:604757. doi: 10.3389/fgene.2020.604757 (PMC7844324; doi:10.3389/fgene.2020.604757)
Supplement: Supplementary Table 1 — Accession numbers of chemosensory genes from other insects used for chemosensory gene identification in A. planipennis. [file Table_1.DOC]

**Table S1 Accession numbers of chemosensory genes from other insects**

| Genes | Accession |
| --- | --- |
| D.mel-CSP A 56a | NP_001027438 |
| D.mel-CSP A 46a | NP_001027399 |
| D.mel-CSP A 86a | NP_001027174 |
| D.mel-CSP A 84a | NP_001027150 |
| D.mel-CSP A 98a | NP_652269 |
| D.mel-CSP B 42c | NP_995764 |
| D.mel-CSP B 93b | NP_732641 |
| D.mel-CSP B 93a | NP_650970 |
| D.mel-CSP A 75a | NP_649035 |
| D.mel-CSP A 7a | NP_572395 |
| D.mel-CSP B 74a | NP_001027134 |
| D.mel-CSP B 53b | NP_001014531 |
| D.mel-CSP B 53a | NP_001014530 |
| D.mel-CSP B 42a | NP_995765 |
| D.mel-CSP B 42b | NP_995763 |
| D.mel-CSP B 38a | NP_995736 |
| D.mel-CSP B 38b | NP_995735 |
| D.mel-CSP A 29a | NP_788004 |
| D.mel-CSP A 87a | NP_650280 |
| D.mel-CSP B 38c | NP_610061 |
| D.mel-GR 97a isoform B | NP_001287551 |
| D.mel-GR 2a isoform C | NP_001162633 |
| D.mel-GR 2a isoform B | NP_525038 |
| D.mel-GR 28b isoform B | NP_995643 |
| D.mel-GR 28b isoform C | NP_995642 |
| D.mel-GR 28b isoform D | NP_995641 |
| D.mel-GR 28b isoform E | NP_995640 |
| D.mel-GR 22c | NP_722732 |
| D.mel-GR 28b isoform A | NP_647614 |
| D.mel-GR 28a | NP_523504 |
| D.mel-GR 97a isoform A | NP_788747 |
| D.mel-GR 59b | NP_523818 |
| D.mel-GR 98c | NP_524531 |
| D.mel-GR 5a | NP_511050 |
| D.mel-GR 61a isoform C | NP_001286892 |
| D.mel-GR 43a isoform C | NP_001286158 |
| D.mel-GR 61a isoform B | NP_001261226 |
| D.mel-GR 22d partial | NP_001259882 |
| D.mel-GR 21a isoform B | NP_001259841 |
| D.mel-GR 47a | NP_523682 |
| D.mel-GR 66a | NP_523971 |
| D.mel-GR 93b | NP_732664 |
| D.mel-GR 63a | NP_001137883 |
| D.mel-GR 23a isoform C | NP_523459 |
| D.mel-GR 43a isoform B | NP_001036531 |
| D.mel-GR 64e isoform A | NP_728923 |
| D.mel-GR 64e isoform B | NP_001027106 |
| D.mel-GR 22b | NP_001014456 |
| D.mel-GR 92a | NP_732489 |
| D.mel-GR 64f | NP_728924 |
| D.mel-GR 58a | NP_726135 |
| D.mel-GR 47b | NP_725040 |
| D.mel-GR 36a | NP_724038 |
| D.mel-GR 22f | NP_722729 |
| D.mel-GR 59d | NP_611758 |
| D.mel-GR 33a | NP_525102 |
| D.mel-GR 43a isoform A | NP_523650 |
| D.mel-GR 32a | NP_523543 |
| D.mel-GR 21a isoform A | NP_001259841 |
| D.mel-GR 23a isoform B | NP_787965 |
| D.mel-GR 58b | NP_523808 |
| D.mel-GR 59e | NP_788431 |
| D.mel-GR 59f | NP_788432 |
| D.mel-GR 58c | NP_726133 |
| D.mel-GR 8a | NP_511097 |
| D.mel-GR 77a | NP_730560 |
| D.mel-GR 68a | NP_524027 |
| D.mel-GR 59c | NP_726292 |
| D.mel-GR 64a | NP_728920 |
| D.mel-GR 98d | NP_733214 |
| D.mel-GR 98b | NP_733213 |
| D.mel-GR 98a | NP_651564 |
| D.mel-GR 94a | NP_732816 |
| D.mel-GR 93d | NP_732666 |
| D.mel-GR 93c | NP_732665 |
| D.mel-GR 93a | NP_524442 |
| D.mel-GR 10a | NP_727523 |
| D.mel-GR 39b | NP_724336 |
| D.mel-GR 39a isoform A | NP_724332 |
| D.mel-GR 39a isoform D | NP_724331 |
| D.mel-GR 39a isoform C | NP_724330 |
| D.mel-GR 39a isoform B | NP_724329 |
| D.mel-GR 36c | NP_724040 |
| D.mel-GR 36b | NP_724039 |
| D.mel-GR 22a | NP_722733 |
| D.mel-GR 22e | NP_722731 |
| D.mel-GR 61a isoform A | NP_001261226 |
| D.mel-GR 57a | NP_523798 |
| D.mel-GR 10b | NP_511121 |
| D.mel-GR 64d isoform C | NP_001027105 |
| D.mel-GR 64c isoform B | NP_001246610 |
| D.mel-GR 64d isoform A | NP_728922 |
| D.mel-GR 59a | NP_726287 |
| D.mel-GR 89a | NP_650555 |
| D.mel-GR 64c isoform A | NP_001246610 |
| D.mel-GR 64b | NP_728921 |
| D.mel-GR 85a | NP_731426 |
| D.mel-GR 9a | NP_727392 |
| D.mel-GR 64e isoform B | XP_015049675 |
| D.mel-GR 64e isoform A | XP_002093625 |
|  |  |
| D.mel-IR 25a isoform E | NP_001260051 |
| D.mel-IR 25a isoform D | NP_001260050 |
| D.mel-IR 25a isoform C | NP_001260049 |
| D.mel-IR 25a isoform B | NP_001260051 |
| D.mel-IR 68a isoform B | NP_001287031 |
| D.mel-IR 40a isoform G | NP_001286132 |
| D.mel-IR 40a isoform F | NP_610140 |
| D.mel-IR 40a isoform E | NP_001260687 |
| D.mel-IR 31a isoform D | NP_723585 |
| D.mel-IR 31a isoform C | NP_001260346 |
| D.mel-IR 7c isoform B | NP_001245572 |
| D.mel-IR 76a isoform E | NP_001097647 |
| D.mel-IR 76a isoform D | NP_001137982 |
| D.mel-IR 68a isoform A | NP_648455 |
| D.mel-IR 76a isoform B | NP_649148/NP_001097647 |
| D.mel-IR 76a isoform A | NP_649148 |
| D.mel-IR 7c isoform A | NP_001245572 |
| D.mel-IR 8a | NP_727328 |
| D.mel-IR 67a | NP_648329 |
| D.mel-IR 60e | NP_611927 |
| D.mel-IR 41a | NP_995744 |
| D.mel-IR 94c | NP_732701 |
| D.mel-IR 94b | NP_732700 |
| D.mel-IR 93a | NP_650924 |
| D.mel-IR 92a | NP_001097845 |
| D.mel-IR 87a | NP_650290 |
| D.mel-IR 84a | NP_649720 |
| D.mel-IR 11a | NP_572795 |
| D.mel-IR 7b | NP_572410 |
| D.mel-IR 75d | NP_649074 |
| D.mel-IR 75c | NP_649013 |
| D.mel-IR 75b | NP_001137966 |
| D.mel-IR 60d | NP_001137757 |
| D.mel-IR 75a | NP_649012 |
| D.mel-IR 60b | NP_001137755 |
| D.mel-IR 94h | NP_651148 |
| D.mel-IR 94g | NP_651147 |
| D.mel-IR 94f | NP_732868 |
| D.mel-IR 94e | NP_001097885 |
| D.mel-IR 94d | NP_001138099 |
| D.mel-IR 54a | NP_611259 |
| D.mel-IR 52d | NP_611042 |
| D.mel-IR 52b | NP_725469 |
| D.mel-IR 52a | NP_611041 |
| D.mel-IR 7g | NP_001368933 |
| D.mel-IR 7f | NP_001138177 |
| D.mel-IR 7e | NP_001138176 |
| D.mel-IR 7d | NP_001138175 |
| D.mel-IR 10a partial | NP_001096949 |
| D.mel-IR 21a | NP_001097043 |
| D.mel-IR 62a | NP_001033986 |
| D.mel-IR 100a | NP_651898 |
| D.mel-IR 60a | NP_611901 |
| D.mel-IR 76b | NP_649176 |
| D.mel-IR 68b | NP_648548 |
| D.mel-IR 67c | NP_729609 |
| D.mel-IR 67b | NP_648393 |
| D.mel-IR 64a | NP_647962 |
| D.mel-IR 56d | NP_611432 |
| D.mel-IR 56c | NP_611431 |
| D.mel-IR 56b | NP_611430 |
| D.mel-IR 56a | NP_725850 |
| D.mel-IR 52c | NP_725470 |
| D.mel-IR 51b | NP_725440 |
| D.mel-IR 48c | NP_610700 |
| D.mel-IR 48b | NP_610697 |
| D.mel-IR 47a | NP_610580 |
| D.mel-IR 94a | NP_732699 |
| D.mel-IR 85a | NP_649833 |
| D.mel-IR 20a | NP_608456 |
| D.mel-IR 7a | NP_572406 |
|  |  |
| D.mel-OBP 83cd | NP_649612 |
| D.mel-OBP 99b isoform B | NP_001263078 |
| D.mel-OBP 99b isoform A | NP_001287586 |
| D.mel-OBP 99a isoform B | NP_001287586 |
| D.mel-OBP 19c isoform C | NP_608392 |
| D.mel-OBP 99a isoform A | NP_001287586 |
| D.mel-OBP 19c isoform A | NP_608392 |
| D.mel-OBP 83ef | NP_731042 |
| D.mel-OBP 56e isoform B | NP_001286620 |
| D.mel-OBP 58b | NP_611709 |
| D.mel-OBP 56e isoform A | NP_611445 |
| D.mel-OBP 84a isoform C | NP_001097700 |
| D.mel-OBP 28a | NP_523505 |
| D.mel-OBP 84a isoform A | NP_476990 |
| D.mel-OBP 83a isoform C | NP_001287190 |
| D.mel-OBP 83a isoform B | NP_001287189 |
| D.mel-OBP 83a isoform A | NP_001287190 |
| D.mel-OBP 56h isoform B | NP_001188979 |
| D.mel-OBP 56h isoform A | NP_001188979 |
| D.mel-OBP 56b | NP_611443 |
| D.mel-OBP 18a | NP_573350 |
| D.mel-OBP 8a | NP_727322 |
| D.mel-OBP 57e | NP_611488 |
| D.mel-OBP 57c | NP_611481 |
| D.mel-OBP 44a isoform B | NP_001286186 |
| D.mel-OBP 19d isoform B | NP_523421 |
| D.mel-OBP 69a | NP_524039 |
| D.mel-OBP 57a | NP_725966 |
| D.mel-OBP 83b | NP_524242 |
| D.mel-OBP 19d isoform A | NP_523421 |
| D.mel-OBP 44a isoform A | NP_001286186 |
| D.mel-OBP 73a isoform C | NP_001334711 |
| D.mel-OBP 85a | NP_001334691 |
| D.mel-OBP 57b isoform B | NP_001286632 |
| D.mel-OBP 56d isoform B | NP_001286619 |
| D.mel-OBP 50c | NP_725387 |
| D.mel-OBP 22a isoform C | NP_001014457 |
| D.mel-OBP 99c isoform B | NP_001263077 |
| D.mel-OBP 56c isoform C | NP_725925 |
| D.mel-OBP 19a | NP_728338 |
| D.mel-OBP 56i isoform B | NP_001188980 |
| D.mel-OBP 73a isoform B | NP_001097628 |
| D.mel-OBP 22a isoform B | NP_001014457 |
| D.mel-OBP 56g isoform B | NP_995903 |
| D.mel-OBP 56c isoform B | NP_995902 |
| D.mel-OBP 50a isoform B | NP_995832 |
| D.mel-OBP 47a isoform B | NP_995810 |
| D.mel-OBP 50d | NP_725388 |
| D.mel-OBP 50e | NP_610959 |
| D.mel-OBP 59a | NP_788429 |
| D.mel-OBP 58d | NP_611711 |
| D.mel-OBP 58c | NP_611710 |
| D.mel-OBP 57d | NP_725973 |
| D.mel-OBP 57b isoform A | NP_001286632 |
| D.mel-OBP 56i isoform A | NP_725929 |
| D.mel-OBP 56g isoform A | NP_611447 |
| D.mel-OBP 56f | NP_725926 |
| D.mel-OBP 56d isoform A | NP_001286619 |
| D.mel-OBP 51a | NP_725436 |
| D.mel-OBP 50b | NP_725386 |
| D.mel-OBP 50a isoform A | NP_725385 |
| D.mel-OBP 49a | NP_610812 |
| D.mel-OBP 47b | NP_610669 |
| D.mel-OBP 47a isoform A | NP_610632 |
| D.mel-OBP 46a | NP_610574 |
| D.mel-OBP 99d | NP_651712 |
| D.mel-OBP 99c isoform A | NP_651711 |
| D.mel-OBP 93a | NP_650945 |
| D.mel-OBP 83g | NP_731043 |
| D.mel-OBP 19b | NP_608391 |
| D.mel-OBP 56a | NP_611442 |
|  |  |
| D.mel-OR 22b | NP_477425 |
| D.mel-OR 7a | NP_511081 |
| D.mel-OR 22a | NP_523453 |
| D.mel-OR 33a | NP_523553 |
| D.mel-OR 33b | NP_523554 |
| D.mel-OR 22c | NP_523454 |
| D.mel-OR 43b | NP_523656 |
| D.mel-OR 42b | NP_523624 |
| D.mel-OR 42a | NP_523622 |
| D.mel-OR 85f | NP_524289 |
| D.mel-OR 85d | NP_524281 |
| D.mel-OR 74a | NP_524123 |
| D.mel-OR 49b | NP_523721 |
| D.mel-OR 45b | NP_523667 |
| D.mel-OR 10a | NP_511122 |
| D.mel-OR 9a | NP_511107 |
| D.mel-OR 2a | NP_525046 |
| D.mel-OR 94b | NP_524456 |
| D.mel-OR 94a | NP_524455 |
| D.mel-OR 85a | NP_524277 |
| D.mel-OR 47a | NP_523689 |
| D.mel-OR 59c | NP_523823 |
| D.mel-OR 59b | NP_523822 |
| D.mel-OR 59a | NP_523821 |
| D.mel-OR 33c | NP_523555 |
| D.mel-OR 67a | NP_524005 |
| D.mel-OR 65a | NP_729161 |
| D.mel-OR 63a isoform A | NP_523895 |
| D.mel-OR 56a | NP_523796 |
| D.mel-OR 98a | NP_524536 |
| D.mel-OR 92a | NP_524414 |
| D.mel-OR 88a | NP_524348 |
| D.mel-OR 85c | NP_524280 |
| D.mel-OR 85b | NP_524279 |
| D.mel-OR 83c | NP_524244 |
| D.mel-OR co-receptor isoform A | NP_001097687 |
| D.mel-OR 83a | NP_524234 |
| D.mel-OR 82a | NP_730794 |
| D.mel-OR 19a | NP_525013 |
| D.mel-OR 19b | NP_728315 |
| D.mel-OR 13a | NP_523359 |
| D.mel-OR 1a | NP_525029 |
| D.mel-OR 43a | NP_523647 |
| D.mel-OR 35a | NP_723916 |
| D.mel-OR 30a | NP_523520 |
| D.mel-OR 98b partial | NP_524540 |
| D.mel-OR 85e partial | NP_001262374 |
| D.mel-OR 71a isoform D | NP_001246763 |
| D.mel-OR 63a isoform B | NP_001163331 |
| D.mel-OR 24a | NP_523470 |
| D.mel-OR co-receptor isoform B | NP_001097687 |
| D.mel-OR 65c | NP_729163 |
| D.mel-OR 65b | NP_729162 |
| D.mel-OR 69a isoform A | NP_996070 |
| D.mel-OR 69a isoform B | NP_996069 |
| D.mel-OR 46a isoform A | NP_995794 |
| D.mel-OR 46a isoform B | NP_995793 |
| D.mel-OR 67d | NP_648390 |
| D.mel-OR 47b | NP_523690 |
| D.mel-OR 23a | NP_523458 |
| D.mel-OR 49a | NP_523711 |
| D.mel-OR 45a | NP_523666 |
| D.mel-OR 71a isoform B | NP_524078 |
| D.mel-OR 67c | NP_524018 |
| D.mel-OR 67b | NP_524007 |
|  |  |
| D.mel-SNMP 1 isoform B | NP_001262803 |
| D.mel-SNMP 1 isoform A | NP_001262803 |
| D.mel-SNMP 2 isoform D | NP_001163372 |
| D.mel-SNMP 2 isoform C | NP_001163372 |
| D.mel-SNMP 2 isoform B | NP_001036593 |
|  |  |
| B.mor-CSP 4 precursor | NP_001037052 |
| B.mor-CSP 10 | NP_001037064 |
| B.mor-CSP 13 precursor | NP_001037180 |
| B.mor-CSP 12 precursor | NP_001091780 |
| B.mor-CSP 14 precursor | NP_001037192 |
| B.mor-CSP 6 precursor | NP_001037400 |
| B.mor-CSP 3 precursor | NP_001037063 |
| B.mor-CSP 5 precursor | NP_001037062 |
| B.mor-CSP 7 precursor | NP_001037068 |
| B.mor-CSP 1 precursor | NP_001037065 |
| B.mor-CSP 9 precursor | NP_001037069 |
| B.mor-CSP precursor | NP_001037066 |
| B.mor-CSP 2 precursor | NP_001091778 |
| B.mor-CSP 8 precursor | NP_001037067 |
| B.mor-CSP 11 precursor | NP_001091779 |
| B.mor-CSP 16 precursor | NP_001091782 |
| B.mor-CSP 16 isoform X2 | XP_021205867 |
| B.mor-CSP 16 isoform X1 | XP_021205866 |
| B.mor-CSP 15 isoform X1 | NP_001091781 |
| B.mor-CSP 12 isoform X2 | XP_021206026 |
| B.mor-CSP 12 isoform X2 | XP_021206023 |
| B.mor-CSP 12 isoform X2 | XP_021206021 |
| B.mor-CSP 12 isoform X1 | XP_021206019 |
| B.mor-CSP 11 isoform X2 | XP_012549404 |
| B.mor-CSP 11 isoform X2 | XP_012549404 |
| B.mor-CSP 6 isoform X1 | NP_001037400 |
| B.mor-CSP 1 isoform X1 | NP_001037065 |
| B.mor-CSP 1 isoform X1 | NP_001037065 |
| B.mor-CSP 11 isoform X2 | XP_012549404 |
| B.mor-CSP 11 isoform X2 | XP_012549404 |
| B.mor-CSP 11 isoform X2 | XP_012549404 |
| B.mor-CSP 11 isoform X1 | XP_012549404 |
| B.mor-CSP 6 isoform X1 | NP_001037400 |
| B.mor-CSP 9 isoform X1 | XP_012549310 |
| B.mor-CSP isoform X1 | XP_012549269 |
| B.mor-CSP 1 isoform X1 | NP_001037065 |
| B.mor-CSP 10 isoform X1 | XP_012549237 |
| B.mor-CSP 10 isoform X1 | XP_012549237 |
| B.mor-CSP 15 | NP_001091781 |
|  |  |
| B.mor-GR 9 | NP_001124345 |
| B.mor-GR 67 | NP_001233216 |
| B.mor-GR 60 | NP_001124347 |
| B.mor-GR 8 | NP_001124344 |
| B.mor-GR 10 | NP_001091791 |
| B.mor-GR 45 | NP_001124346 |
| B.mor-GR 9 isoform X2 | XP_012551881 |
| B.mor-GR 9 isoform X1 | XP_012551875 |
| B.mor-GR 68 | NP_001233217 |
| B.mor-GR 7 | XP_021208674 |
| B.mor-GR 17 | XP_004923309 |
| B.mor-GR 29 | 相似性小于50％ |
| B.mor-GR 28 | 相似性小于50％ |
| B.mor-GR 27 | 相似性小于50％ |
| B.mor-GR 57 | 相似性小于50％ |
| B.mor-GR 56 | 相似性小于50％ |
| B.mor-GR 53 | XP_004932760 |
| B.mor-GR 51 | 相似性小于50％ |
| B.mor-GR 50 | 相似性小于50％ |
| B.mor-GR 27 | 相似性小于50％ |
| B.mor-GR 18 | 相似性小于50％ |
| B.mor-GR 17 | XP_004923309 |
| B.mor-GR 16 | 相似性小于50％ |
| B.mor-GR 14 | 相似性小于50％ |
| B.mor-GR 11 | NONE |
| B.mor-GR 9 | NP_001124345 |
| B.mor-GR 8 | NP_001124344 |
| B.mor-GR 31 | 相似性小于50％ |
| B.mor-GR 30-8 | 相似性小于50％ |
| B.mor-GR sugar taste 64f-like | XP_021208674 |
| B.mor-GR sugar taste 64f-like | XP_021209042 |
| B.mor-GR sugar taste 64a | XP_021208998 |
| B.mor-GR 22 | XP_004932263 |
| B.mor-GR 24 | XP_004931409 |
| B.mor-GR 28a | XP_012546109 |
| B.mor-GR 22 | XP_004932263 |
| B.mor-GR 47 partial | XP_004928010 |
| B.mor-GR 46 partial | XP_004927812 |
| B.mor-GR 68 | NP_001233217 |
| B.mor-GR 67 | NP_001233216 |
| B.mor-GR 66 | XP_012546109 |
| B.mor-GR 64 | 相似性小于50％ |
| B.mor-GR 63 | XP_012550875 |
| B.mor-GR 62 | XP_021204387 |
| B.mor-GR 61 | 相似性小于50％ |
| B.mor-GR 58 | 相似性小于50％ |
| B.mor-GR 33 | NONE |
| B.mor-GR 30 | 相似性小于50％ |
| B.mor-GR 29 | 相似性小于50％ |
| B.mor-GR 26 | 相似性小于50％ |
| B.mor-GR 15 | XP_004923309 |
| B.mor-GR 13 | 相似性小于50％ |
| B.mor-GR 60 | NP_001124347 |
| B.mor-GR 45 | NP_001124346 |
|  |  |
| B.mor-IR 68a | XP_021206441 |
| B.mor-IR 41a | XP_012550734 |
| B.mor-IR 21a | XP_021207769 |
| B.mor-IR 64a | XP_021205850 |
| B.mor-IR 75d | XP_021204132 |
| B.mor-IR 75p | XP_021208462 |
| B.mor-IR 75q1 | XP_021203485 |
| B.mor-IR 75q2 | XP_021203484 |
| B.mor-IR 40a | XP_021202684 |
| B.mor-IR 76b | XP_004927781 |
| B.mor-IR 93a | XP_021203225 |
| B.mor-IR 8a | XP_012547019 |
| B.mor-IR 25a | XP_021207886 |
|  |  |
| B.mor-OBP LOC100301495 | NP_001153663 |
| B.mor-OBP fmxg18C17 | NP_001157372 |
| B.mor-OBP LOC100307012 | NP_001159621 |
| B.mor-OBP LOC100301497 | NP_001153665 |
| B.mor-OBP LOC100307013 | NP_001153664 |
| B.mor-OBP LOC100301496 | NP_001153664 |
| B.mor-OBP 3 precursor | NP_001077095 |
| B.mor-OBP 3 isoform X1 | XP_021203662 |
| B.mor-OBP precursor | NP_001037494 |
| B.mor-OBP 2 precursor | NP_001037498 |
| B.mor-OBP 1 precursor | NP_001037496 |
| B.mor-OBP 5 precursor | NP_001140189 |
| B.mor-OBP 7 precursor | NP_001140191 |
| B.mor-OBP 6 | NP_001140190 |
| B.mor-OBP 2 precursor | NP_001140186 |
| B.mor-OBP 3 precursor | NP_001140187 |
| B.mor-OBP 1 | NP_001140185 |
| B.mor-OBP 4 | NP_001140188 |
| B.mor-OBP 1 isoform X1 | XP_021204926 |
| B.mor-OBP 6 isoform X1 | XP_012547442 |
| B.mor-OBP 4 isoform X1 | XP_012547441 |
| B.mor-OBP | XP_012545845 |
|  |  |
| B.mor-OR 12 isoform X1 | XP_021207677 |
| B.mor-OR 12 | NP_001116806 |
| B.mor-OR 4 | XP_021208172 |
| B.mor-OR 13a-like | XP_021206913 |
| B.mor-OR 43a-like | XP_012548773 |
| B.mor-OR 85d | XP_021205839 |
| B.mor-OR 4 | XP_021205480 |
| B.mor-OR 4 isoform X3 | XP_021205151 |
| B.mor-OR 4 isoform X2 | XP_021205150 |
| B.mor-OR 4 isoform X1 | XP_012547825 |
| B.mor-OR Or1-like | XP_021204320 |
| B.mor-OR 4-like | XP_021203888 |
| B.mor-OR 4 | XP_021203284 |
| B.mor-OR 4 | XP_021203274 |
| B.mor-OR 49b | XP_021203152 |
| B.mor-OR 85e | NP_001116817 |
| B.mor-OR 56a-like | XP_004934169 |
| B.mor-OR 4 isoform X1 | XP_012547825 |
| B.mor-OR 85d | XP_012547463 |
| B.mor-OR 94b | XP_012545300 |
| B.mor-OR 4 | XP_004928758 |
| B.mor-OR 10 | NP_001104819 |
| B.mor-OR 47 | NP_001104818 |
| B.mor-OR 45 | NP_001104798 |
| B.mor-OR 7 isoform X1 | XP_021204088 |
| B.mor-OR 29 | NP_001166894 |
| B.mor-OR 23 | NP_001166606 |
| B.mor-OR-like | NP_001159623 |
| B.mor-OR 24 | NP_001155300 |
| B.mor-OR 18 | NP_001166895 |
| B.mor-OR 27 | NP_001166893 |
| B.mor-OR 11 | NP_001166604 |
| B.mor-OR 39 | NP_001116807 |
| B.mor-OR 9 | NP_001116805 |
| B.mor-OR 7 | NP_001106227 |
| B.mor-OR 37 | NP_001104799 |
| B.mor-OR 33 | NP_001103623 |
| B.mor-OR 38 | NP_001103477 |
| B.mor-OR 35 | NP_001103476 |
| B.mor-OR 42 | NP_001091818 |
| B.mor-OR 19 | NP_001091785 |
| B.mor-OR 2 | NP_001037060 |
| B.mor-OR 3 | NP_001036925 |
| B.mor-OR 4 | NP_001036926 |
| B.mor-OR-like | NP_001116810 |
| B.mor-OR 1 | NP_001036875 |
| B.mor-OR 4 isoform X1 | XP_021208181 |
| B.mor-OR 9 isoform X1 | XP_021208177 |
| B.mor-OR 65 isoform X1 | XP_012550102 |
| B.mor-OR 55 isoform X1 | XP_021206373 |
| B.mor-OR 6 isoform X1 | XP_021205710 |
| B.mor-OR-like isoform X2 | XP_021205365 |
| B.mor-OR 10 isoform X2 | XP_021203668 |
| B.mor-OR 10 isoform X1 | XP_021203667 |
| B.mor-OR 45 isoform X1 | XP_021203125 |
| B.mor-OR 17 isoform X1 | XP_021202897 |
| B.mor-OR 60 isoform X1 | XP_021202210 |
| B.mor-OR 59 isoform X2 | XP_021202108 |
| B.mor-OR 59 isoform X1 | XP_021202107 |
| B.mor-OR 11 isoform X1 | XP_021209282 |
| B.mor-OR 49 isoform X1 | XP_012549130 |
| B.mor-OR 16 isoform X2 | NP_001166608 |
| B.mor-OR 16 isoform X1 | XP_021202205 |
| B.mor-OR 53 isoform X1 | XP_012550237 |
| B.mor-OR 44 isoform X1 | XP_012548737 |
| B.mor-OR 2 isoform X1 | XP_012548069 |
| B.mor-OR-like isoform X1 | XP_012548049 |
| B.mor-OR 63 isoform X2 | XP_012545176 |
| B.mor-OR 63 isoform X1 | XP_012545175 |
| B.mor-OR 13 | NP_001166603 |
| B.mor-OR 41 | NP_001091787 |
| B.mor-OR 56 | NP_001166617 |
| B.mor-OR 54 | NP_001166616 |
| B.mor-OR 59 | NP_001166611 |
| B.mor-OR 14 | NP_001166602 |
| B.mor-OR 8 | NP_001157209 |
| B.mor-OR 60 | NP_001155301 |
| B.mor-OR 64 | NP_001166621 |
| B.mor-OR 17 | NP_001157210 |
| B.mor-candidate OR | NP_001091790 |
| B.mor-OR 36 | NP_001166892 |
| B.mor-OR 44 | NP_001166607 |
| B.mor-OR 65 | NP_001166622 |
| B.mor-OR 46 | NP_001155299 |
| B.mor-OR 25 | NP_001104828 |
| B.mor-OR 15 | NP_001091789 |
| B.mor-OR 16 | NP_001104832 |
| B.mor-OR 30 | NP_001091786 |
| B.mor-OR 21 | NP_001104831 |
| B.mor-OR 12 | NP_001104829 |
| B.mor-OR 34 | NP_001103624 |
| B.mor-OR 5 | NP_001036927 |
| B.mor-OR 40 | NP_001166608 |
| B.mor-OR 20 | NP_001166605 |
| B.mor-OR 22 | NP_001166613 |
| B.mor-OR 53 | NP_001166615 |
| B.mor-OR 55 | NP_001166612 |
| B.mor-OR 57 | NP_001159625 |
| B.mor-OR-like | NP_001116817 |
| B.mor-candidate OR | NP_001166602 NP_001091792 |
| B.mor-OR 6 | NP_001036928 |
| B.mor-OR 63 | NP_001166620 |
| B.mor-OR 49 | NP_001166614 |
| B.mor-OR 61 | NP_001166619 |
| B.mor-OR 58 | NP_001166618 |
|  |  |
| B.mor-SNMP 1 | NP_001037186.1 |
| B.mor-SNMP 1-like | XP_012550444 |
| B.mor-SNMP 2 | XP_012547405 |
| B.mor-SNMP 2 | XP_012547405 |
|  |  |
| S.lit-OR 46a-like | XP_022816642 |
| S.lit-OR 4-like | XP_022816228 |
| S.lit-OR 94b-like | XP_022816227 |
| S.lit-OR Or2-like | XP_022815666 |
| S.lit-OR 4-like | XP_022815660 |
| S.lit-OR 85c-like | XP_022814908 |
| S.lit-OR 43a-like | XP_022813974 |
| S.lit-OR Or1-like | XP_022838105 |
| S.lit-OR 4-like | XP_022837644 |
| S.lit-OR Or2-like | XP_022837202 |
| S.lit-OR 83a-like isoform X2 | XP_022831788 |
| S.lit-OR 85e isoform X1 | XP_022831787 |
| S.lit-OR 85c-like | XP_022831643 |
| S.lit-OR coreceptor | XP_022831582 |
| S.lit-OR 4-like | XP_022829617 |
| S.lit-OR 30a-like | XP_022827581 |
| S.lit-OR 4-like | XP_022827447 |
| S.lit-OR 4-like | XP_022826965 |
| S.lit-OR 67c-like | XP_022826805 |
| S.lit-OR 94a-like | XP_022826289 |
| S.lit-OR 67a-like | XP_022825610 |
| S.lit-OR 13a-like | XP_022825580 |
| S.lit-OR 85c-like | XP_022825194 |
| S.lit-OR 13a-like isoform X3 | XP_022825111 |
| S.lit-OR 13a-like isoform X2 | XP_022825110 |
| S.lit-OR 13a-like isoform X1 | XP_022825109 |
| S.lit-OR 49a-like | XP_022824838 |
| S.lit-OR 67c-like | XP_022824679 |
| S.lit-OR 46a-like | XP_022824662 |
| S.lit-OR 85c-like | XP_022826861 |
| S.lit-OR 4-like isoform X3 | XP_022826481 |
| S.lit-OR 4-like isoform X2 | XP_022826473 |
| S.lit-OR 4-like isoform X1 | XP_022826464 |
| S.lit-OR 30a-like | XP_022824106 |
| S.lit-OR 13a-like | XP_022823691 |
| S.lit-OR 13a-like | XP_022823690 |
| S.lit-OR 13a-like isoform X2 | XP_022823505 |
| S.lit-OR 13a-like isoform X1 | XP_022823503 |
| S.lit-OR 4-like | XP_022822473 |
| S.lit-OR 4-like | XP_022822458 |
| S.lit-OR 2a-like | XP_022822183 |
| S.lit-OR 67c-like | XP_022818084 |
| S.lit-OR 67c-like | XP_022818084 |
| S.lit-OR 46a-like | XP_022817447 |
| S.lit-OR Or1-like | XP_022817446 |
| S.lit-OR 10a-like | XP_022816679 |
| S.lit-OR 49b-like partial | XP_022834239 |
| S.lit-LOC111361982 | XP_022834223 |
| S.lit-LOC111362088 | XP_022834383 |
| S.lit-LOC111351909 | XP_022819893 |
| S.lit-LOC111351947 | XP_022819946 |
| S.lit-LOC111351948 | XP_022819948 |
| S.lit-LOC111353873 | XP_022822842 |
| S.lit-LOC111353938 | XP_022822928 |
| S.lit-LOC111353955 | XP_022822952 |
| S.lit-LOC111354009 | XP_022823032 |
| S.lit-LOC111354335 | XP_022823520 |
| S.lit-LOC111355246 | XP_022824791 |
| S.lit-LOC111355465 | XP_022825134 |
| S.lit-LOC111355467 | XP_022825135 |
| S.lit-LOC111355689 | XP_022825487 |
| S.lit-LOC111355779 | XP_022825609 |
| S.lit-LOC111355789 | XP_022825618 |
| S.lit-LOC111362302 | XP_022834730 |
| S.lit-LOC111355511 | XP_022825207 |
| S.lit-OR 22 | XP_022828173 |
| S.lit-LOC111364565 | XP_022837238 |
| S.lit-LOC111355712 | XP_022825517 |
| S.lit-LOC111352887 | XP_022821343 |
| S.lit-LOC111360969 | XP_022833042 |
| S.lit-LOC111349337 | XP_022816198 |
| S.lit-LOC111348556 | XP_022814981 |
| S.lit-LOC111364584 | XP_022837259 |
| S.lit-LOC111362329 | XP_022834763 |
| S.lit-LOC111362319 | XP_022834752 |
| S.lit-LOC111362312 | XP_022834740 |
