# Supplementary material for: The Characteristics of Chemosensory and Opsin Genes in Newly Emerged and Sexually Mature Agrilus planipennis, an Important Quarantine Forest Beetle
Source: Front Genet. 2021 Jan 15;11:604757. doi: 10.3389/fgene.2020.604757 (PMC7844324; doi:10.3389/fgene.2020.604757)
Supplement: Supplementary Table 2 — Accession numbers of opsins from other insects used for opsin gene identification in A. planipennis. [file Table_2.DOC]

**Table S2 Accession numbers of opsins from other insects**

| Genes | Accession |
| --- | --- |
| D.pun-Opsin blue | QIC54073 |
| D.pun-Opsin UV | QIC54074 |
| D.pun-Opsin UV like | QIC54075 |
| D.pun-Opsin LW | QIC54076 |
| T.den-Opsin blue | XP_014224032 |
| T.den-Opsin UV | CAB0032994 |
| T.den-Opsin LW1 | XP_014229778 |
| T.den-Opsin LW2 | XP_014220576 |
| N.vit-Opsin LW1 | NP_001164379 XP_001606063 |
| N.vit-Opsin UV | XP_001608074 |
| N.vit-Opsin blue | XP_001604622 |
| D.mel-Rh1 | XP_002043271 |
| D.mel-Rh2 | NP_524398 |
| D.mel-Rh3 | NP_524411 |
| D.mel-Rh4 | AAA28856 |
| D.mel-Rh5 | NP_001285859 |
| D.mel-Rh6 | NP_524368 |
| A.mel-Opsin UV | NP_001011605 XP_392791 |
| A.mel-Opsin blue | NP_001011606 XP_392042 |
| A.mel-Opsin LW | Q17053 |
| C.abd-Opsin LW | XP_011258142 |
| C.abd-Opsin UV | AAC05092 |
| C.bom-Opsin LW | Q17296 |
| C.bom-Opsin UV | AAC05091 |
| M.sex-opsin 3 | XP_030029364 |
| M.sex-opsin 2 | XP_030037238 |
| M.sex-opsin 1 | XP_030032517 |
| P.gla-Rh1 | AAD34220 |
| P.gla-Rh2 | AAD34221 |
| P.gla-Rh3 | AAD29445 |
| P.gla-Rh4 | AAD34224 |
| P.gla-Rh5 | AAD34222 |
| P.gla-Rh6 | AAD34223 |
| P.xut-RH1 | BAA31721 |
| P.xut-RH2 | NP_001299732 XP_013166629 |
| P.xut-RH3 | BAA31723 |
| S.gre-opsin 1 | Q94741 |
| S.gre-opsin 2 | Q26495 |
| B.imp-Opsin LWRh1 | XP_012237087 |
| B.imp-Opsin LWRh2 | AAS55406 |
| B.ter-Opsin LWRh1 | XP_012172197 |
| B.ter-Opsin LWRh2 | AAS55405 |
| D.aff-Opsin LWRh1 | AAS55403 |
| D.aff-Opsin LWRh2 | AAS55408 |
| D.rin-Opsin LWRh1 | AAS55404 |
| D.rin-Opsin LWRh2 | AAS55407 |
| O.ruf-Opsin LW1 | AAT73202 |
| O.ruf-Opsin LW2 | AAT73203 |
| S.amp-Opsin UV2 | ANN11843 |
| S.amp-Opsin UV1 | APY20668 |
| S.amp-Opsin LW2 | ANN11841 |
| S.amp-Opsin LW1 | ANN11840 |
| C.sau-Opsin UV1 | ANN11834 |
| C.sau-Opsin LW2 | ANN11833 |
| C.sau-Opsin LW1 | ANN11832 |
