# Supplementary material for: The Characteristics of Chemosensory and Opsin Genes in Newly Emerged and Sexually Mature Agrilus planipennis, an Important Quarantine Forest Beetle
Source: Front Genet. 2021 Jan 15;11:604757. doi: 10.3389/fgene.2020.604757 (PMC7844324; doi:10.3389/fgene.2020.604757)
Supplement: Supplementary Table 3 — The expression levels and significance of differentially expressed genes (DEGs) in female A. planipennis. [file Table_3.DOCX]

**Table S3 The expression levels and significance of differentially expressed genes (DEGs) in female *Agrilus planipennis***

|  | OBP7 | OBP10 | OBP5 | CSP3 | CSP1 | CSP8 | CSP22 | CSP11 | CSP12 | CSP4 | GR8NTE |
| --- | --- | --- | --- | --- | --- | --- | --- | --- | --- | --- | --- |
| EF1 | 417.18 | 502.72 | 26.06 | 130.08 | 4671.93 | 19.28 | 979.82 | 1.13 | 7418 | 160.49 | 0.09 |
| EF2 | 628.16 | 742.74 | 26.34 | 307.15 | 7879.17 | 23.55 | 1611.33 | 2.58 | 6120.43 | 218.59 | 0 |
| EF3 | 670.18 | 736.27 | 24.99 | 245.62 | 6864.43 | 27.68 | 1677.46 | 2.42 | 5222.11 | 259.37 | 0.1 |
| MF1 | 301.38 | 157.45 | 57.61 | 103.17 | 4118.01 | 16.31 | 898.89 | 0.72 | 14502.29 | 535.01 | 0.7 |
| MF2 | 290.88 | 173.04 | 89.94 | 92.45 | 2944.36 | 11.68 | 955.36 | 1.2 | 16854.61 | 400.19 | 0.67 |
| MF3 | 268.4 | 129.44 | 64.47 | 84.07 | 4724.31 | 8.98 | 670.07 | 0.75 | 17434.75 | 453.54 | 0.97 |
| MF3 | 220.91 | 229.66 | 75.13 | 150.11 | 4502.13 | 17.7 | 864.66 | 0.59 | 17086.6 | 885.28 | 1.02 |
| *P* value | 0.007 | 0.001 | 0.003 | 0.05 | 0.048 | 0.026 | 0.035 | 0.032 | 0 | 0.042 | 0.001 |

EF: Eclosion-Female; EM: Eclosion-Male; MF: Mating-Females; MM: Mating-Males.
