# Supplementary material for: The Characteristics of Chemosensory and Opsin Genes in Newly Emerged and Sexually Mature Agrilus planipennis, an Important Quarantine Forest Beetle
Source: Front Genet. 2021 Jan 15;11:604757. doi: 10.3389/fgene.2020.604757 (PMC7844324; doi:10.3389/fgene.2020.604757)
Supplement: Supplementary Table 4 — The expression levels and significance of differentially expressed genes (DEGs) in male A. planipennis. [file Table_4.DOCX]

|  | OBP7 | OBP10 | OBP5 | CSP1 | CSP12 | CSP4 | OR16 | OR47 | OR25CTE |
| --- | --- | --- | --- | --- | --- | --- | --- | --- | --- |
| EM1 | 561.76 | 535.33 | 27.96 | 7466.9 | 7886.71 | 169.2 | 0.44 | 0 | 0 |
| EM2 | 449.18 | 433.42 | 17.94 | 6750.46 | 5673.17 | 125.77 | 0.28 | 0.08 | 0 |
| EM3 | 782.29 | 704.87 | 24.25 | 9089.52 | 7109.72 | 259.25 | 0.41 | 0 | 0 |
| MM1 | 250.39 | 128.92 | 86.6 | 2252.05 | 18708.75 | 405.28 | 0.77 | 0.17 | 0.27 |
| MM2 | 252.86 | 105.14 | 75.34 | 2929.5 | 28399.23 | 604.02 | 0.91 | 0.17 | 0.11 |
| MM3 | 348.81 | 138.94 | 75.42 | 2543.55 | 20001.34 | 548.44 | 0.65 | 0.25 | 0.11 |
| *P* | 0.038 | 0.006 | 0 | 0.002 | 0.008 | 0.009 | 0.011 | 0.011 | 0.038 |

**Table S4 The expression levels and significance of differentially expressed genes (DEGs) in male *A. planipennis***

EF: Eclosion-Female; EM: Eclosion-Male; MF: Mating-Females; MM: Mating-Males.
