# Supplementary material for: The Characteristics of Chemosensory and Opsin Genes in Newly Emerged and Sexually Mature Agrilus planipennis, an Important Quarantine Forest Beetle
Source: Front Genet. 2021 Jan 15;11:604757. doi: 10.3389/fgene.2020.604757 (PMC7844324; doi:10.3389/fgene.2020.604757)
Supplement: Supplementary Table 5 — Primers used for validation of RNA-seq data by qPCR. [file Table_5.DOC]

**Table S5 Primers used for validation of RNA-seq data by qPCR.**

| Genes | Sequence (5'—3') |
| --- | --- |
| AplaOBP1FIX-F | TTGCCGATGACGATAGATT |
| AplaOBP1FIX-R | GCCACATGCCCTTATACTT |
| AplaOBP3FIX-F | TAGCTGCTGTGCTGTTGTT |
| AplaOBP3FIX-R | TTATCAGGAGTAAGCCATT |
| AplaOBP6FIX-F | TCCGACAAACTGATAGAAA |
| AplaOBP6FIX-R | CAGGAGGTAAAGTGGAAAT |
| AplaOBP7-F | TGTAATTGCCACAGTTCAG |
| AplaOBP7-R | TCGGGTGTAATAAATCCAG |
| AplaOBP10-F | CGTCGTTGACGAAGAAAGC |
| AplaOBP10-R | AGTGCGAATGCTGGTGGTT |
| AplaOBP12-F | GCCTTGCCTAGAAAGAACC |
| AplaOBP12-R | ATTACGTCGAGAAATCCGT |
| Apla\Orco-F | AGGCATAAGCATGTCGTAA |
| Apla\Orco-R | CATAGACCAGGTAACCCAGTA |
| AplaOR9-F | AAACCCCGAAATCAAACAT |
| AplaOR9-R | AAAACTGAGGCAGGCGAAT |
| AplaOR16-F | CCGTGAATGAAATGAATGG |
| AplaOR16-R | AACATCATCAAGTGGCATAG |
| LWopsin1-F | TCAGCACCACCAAACACTTA |
| LWopsin1-R | CGCTTAGACCCTTTACGATA |
| UVopsin2-F | GAGTAATCAAGCCCAAGC |
| UVopsin2-R | GCGACGCATTTACAGAA |
| ApEF1-α F | CATTGAAACCTACGTTGTCGC |
| ApEF1-α R | ACTGGAGTGCTTAAACCTGG |
